# The RNA helicase, eIF4A‐1, is required for ovule development and cell size homeostasis in Arabidopsis

**DOI:** 10.1111/tpj.13062

**Published:** 2015-12-07

**Authors:** Maxwell S. Bush, Natalie Crowe, Tao Zheng, John H. Doonan

**Affiliations:** ^1^ Department of Cell and Developmental BiologyJohn Innes CentreColney LaneNorwichNR4 7UHUK; ^2^ Institute of Biological, Environmental & Rural SciencesAberystwyth UniversityGogerddan CampusAberystwythSY23 3EEUK; ^3^ Biomedical Research CentreUniversity of East AngliaNorwichNR4 7TJUK; ^4^ Institute of Virology and BiotechnologyZhejiang Academy of Agricultural ScienceHangzhou CityZhejiang Province310021China

**Keywords:** cell cycle, plant growth, translation factor, RNA helicase, DEAD‐box helicase, cell size homeostasis, *Arabidopsis thaliana*

## Abstract

eIF4A is a highly conserved RNA‐stimulated ATPase and helicase involved in the initiation of mRNA translation. The Arabidopsis genome encodes two isoforms, one of which (eIF4A‐1) is required for the coordination between cell cycle progression and cell size. A T‐DNA mutant *eif4a1* line, with reduced eIF4A protein levels, displays slow growth, reduced lateral root formation, delayed flowering and abnormal ovule development. Loss of eIF4A‐1 reduces the proportion of mitotic cells in the root meristem and perturbs the relationship between cell size and cell cycle progression. Several cell cycle reporter proteins, particularly those expressed at G2/M, have reduced expression in *eif4a1* mutant meristems. Single *eif4a1* mutants are semisterile and show aberrant ovule growth, whereas double *eif4a1 eif4a2* homozygous mutants could not be recovered, indicating that eIF4A function is essential for plant growth and development.

## Significance Statement

While transcriptional control mechanisms in cell cycle progression and development have been extensively dissected, less is known about the role of translation. Here we show that an *Arabidopsis* TDNA mutant with reduced levels of the translation initiation factor eIF4A exhibits several growth defects suggesting that eIF4A‐1 is required for normal cell cycle progression and coordination of cell division with cell growth.

## Introduction

Cell growth and division require gene transcription and transcript translation to be coordinated with the cell cycle, so that the correct regulatory and structural proteins are available as and when they are required. Whereas transcriptional control mechanisms have been extensively dissected, much less is known about the role of translation in cell cycle progression and development. Changes in translation can lead to specific developmental abnormalities, such as cancer in animals (Lazaris‐Karatzas *et al*., 1990), and altered organ morphology in plants (Byrne, 2009), suggesting selective or regulated translation contributes to the regulation of different growth processes.

Translation is a complex multistep process, which is probably subject to regulation at a number of levels. Co‐transcriptional processing of the nascent transcript includes the addition of a 5′ m^7^GpppN‐cap and splicing to remove introns, both of which can profoundly alter export to the cytoplasm, the manner of translation as well as the identity of proteins produced. The initiation of the translation step is one of the best‐studied phases, however, and is widely considered to be a key target of regulation (Browning and Bailey‐Serres, 2015). The m^7^GpppN cap on the 5′ end of the transcript, together with the polyA tail, serve as docking points for the binding of several initiation factors in a highly orchestrated manner. Briefly, eIF4E interacts with the 5′ cap of the mRNA and binds the large eIF4G scaffold protein that engages with the eIF4A/eIF4B helicase complex. eIF4A, the prototypical member of a large RNA helicase family, unwinds secondary structures within the mRNA 5′ untranslated region (5′‐UTR), recruiting the *43S* pre‐initiation ribosome complex and allowing it to scan for start codons. Transcripts differ in their 5′‐UTR structure, and therefore eIF4A could be rate‐limiting for transcripts where the 5′‐UTR has a high level of secondary structure. At least two other cytoplasmic complexes in higher plants can bind to the 5′ cap: eIFiso4F, which is composed of eIFiso4E and eIFiso4G (Patrick and Browning, 2012), and 4E homologous protein (Kropiwnicka *et al*., 2015). This diversity of initiation complexes provides further potential for differential translation.

Cap‐dependent translational control is modulated during the mammalian cell cycle, being suppressed during mitosis by eIF4E‐binding proteins (4E‐BP) that prevent eIF4E from binding eIF4G in a phosphorylation‐dependent manner (Gingras *et al*., 1999; Pyronnet *et al*., 2001). An alternative cap‐independent mechanism that uses internal ribosome entry sites (IRES), instead of a complete eIF4F complex, becomes more important during mitosis, and some cell cycle‐related proteins seem to be synthesized by this mechanism (reviewed by Bugaut and Balasubramanian, 2012). Progression into the M‐phase coincides with the extensive phosphorylation of numerous substrates by the cyclin‐dependent kinase 1 (Cdk‐1) cyclin B complex, and as translation initiation is the rate‐limiting step in translation, it is not surprising that eIF4F components are targets of regulation by phosphorylation (reviewed by Browning and Bailey‐Serres, 2015; Pierrat *et al*., 2007). The phosphorylation of eIF4F proteins per se is not as simple a regulatory mechanism as it might at first appear. For instance, in mammalian cells, Cdk1:cyclin B phosphorylation of Ser1232 in eIF4G during mitosis strongly increases the association of eIF4A with the eIF4G HEAT 2 domain, and decreases the RNA binding affinity of that complex. This effectively ‘freezes’ the helicase complex in an inactive ‘idling state’. The opposite effect is seen when Erk1/2 phosphorylates the same Ser1232 residue (Dobrikov *et al*., 2014). Also, phosphorylation at eIF4B residues Ser406/422 and Ser459/46s is absent or decreased during mitosis, whereas Ser214 and Tyr592 are heavily phosphorylated at mitosis (Dobrikov *et al*., 2014).

Flowering plants lack 4E‐BPs, but there is evidence that eIF4A is phosphorylated (Pierrat *et al*., 2007). It is intriguing then that Arabidopsis eIF4A physically interacts with the cell cycle regulator CDKA, the plant orthologue of CDK1 (Hutchins *et al*., 2004), which could provide a mechanism for modulating its activity. Protein phosphorylation of eIF4A has also been observed in mature leaves, where it appears to be modulated in a light/dark cycle manner (Boex‐Fontvieille *et al*., 2013). Alternative regulatory mechanisms are possible and eIF4A associates with at least four distinct cap complexes: eIF4F, CBP20/CBP80, and the plant‐specific eIFiso4F and nCBP (Bush *et al*., 2009). Notably, the interaction between eIF4A and all four complexes appear to be strongest in proliferating cells. A proteomic analysis of cap‐binding complexes indicates that, in quiescent cells, eIF4A is replaced by a variety of other RNA helicases (Bush *et al*., 2009).

The specific role of eIF4A in plant development and growth is poorly understood, however, let alone the effect that phosphorylation of eIF4A might have. We previously reported that mutants in *Brachypodium distachyon* with reduced levels of eIF4A were smaller and could be complemented by the expression of the Arabidopsis *eIF4A1* gene (Vain *et al*., 2011). The reduced plant stature was associated with reduced cell number and size, suggesting a defect in both cell division and growth. This prompted us to examine the role of eIF4A in Arabidopsis growth and development in more detail. We took a genetic approach to identify and characterize lines with reduced eIF4A levels. Arabidopsis has two genes for eIF4A that are involved in translation (At3 g13920, *EIF4A1* and At1 g54270, *EIF4A2*); the two proteins are 96% similar at the primary sequence level. A third related protein, EIF4A3, is part of the exon junction complex (Koroleva *et al*., 2009a,b), and is not believed to be directly involved in translation. We show that one form of eIF4A is required for normal growth and cell cycle progression, as *EIF4A1* knock‐out mutants are slow growing, late flowering and semisterile compared with *EIF4A2* knock‐out mutants, which show no obvious phenotype. Reduction of eIF4A‐1 appears to specifically perturb the relationship between cell cycle progression and growth in a cell type‐specific manner, leading to increased cell size in roots as well as uncoordinated tissue development in ovules.

## Results

### eIF4A is highly expressed in growing tissues

*EIF4A*1 and *EIF4A*2 promoter::GUS fusions indicate that both genes are highly expressed in both root and shoot meristems, as well as other growing tissues (Figure S1), and this is supported by analysis of public microarray data (http://www.genevestigator.ethz.ch; http://bbc.botany.utoronto.ca/efp/cgi-bin/efpWeb.cgi). Gene expression is highest in meristems and flowers, coincident with the regions displaying phenotypes.

### An *eif4a1* insertion mutant reduces the level of eIF4A protein

**Figure 1:**
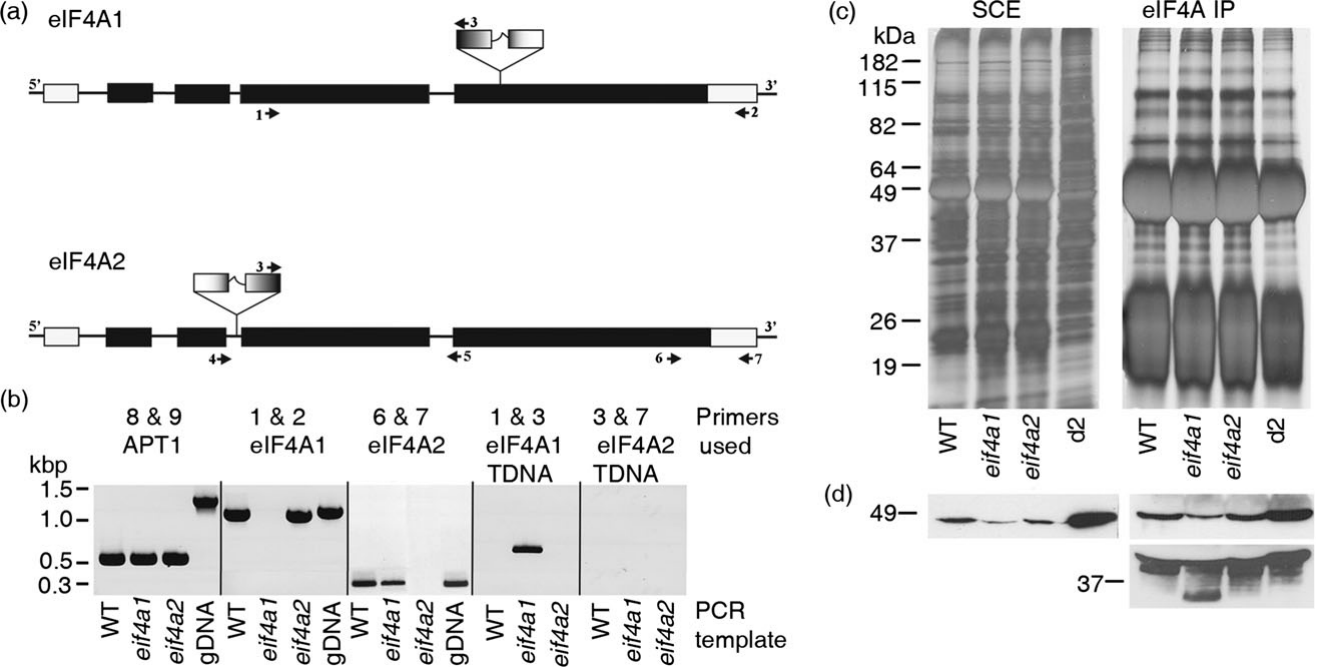
Identification of *eIF4A* insertion mutants. (a) Schematic representation of insertional mutation in *EIF4A1* and *EIF4A2* genes. Primers used for genotyping (Table S1) are indicated by numbered arrowheads. (b) Transcript analysis of eIF4A expression in wild‐type (WT) and mutant strains. RT‐PCRs using cDNA from Arabidopsis Columbia‐0 WT controls and the *eif4a1* and *eif4a2* T‐DNA insertion mutants. The APT1 loading control used primers spanning five introns of the *APT1* gene, confirming that cDNA samples were free of contaminating genomic DNA and equally loaded. The *EIF4A1* transcript is not detectable in the *eif4a1* sample using primers spanning the T‐DNA insertion site, but it is present in the WT and *eif4a2* mutant. In genomic DNA there is a small intron present, and hence the *EIF4A1* gDNA PCR product is slightly larger compared with that of the cDNA products. Likewise, the *EIF4A2* transcript is not detected in the *eif4a2* sample (*EIF4A2*), but is in the *eif4a1* sample. A partial *EIF4A1* transcript from the third exon to the T‐DNA insertion site is detected in the *eif4a1* sample (*EIF4A1* T‐DNA), suggesting the possibility that a truncated eIF4A‐1 protein could be translated. (c, d) Analysis of eIF4A‐1 protein levels in WT and mutant plants. (c) Silver‐stained SDS‐PAGE gels of soluble cell protein extracts (SCE) and anti‐wheat eIF4A immunoprecipitations (eIF4A IP) from Columbia‐0 (WT), *eif4a1* and *eif4a2* plants, and a 2‐day‐old *Arabidopsis* cell culture (d2) as an internal control. The band intensities indicate similar protein loadings for all plant samples. Duplicate gels were immunoblotted with the anti‐wheat eIF4A antibody (d). (d) Western‐blot analysis of eIF4A levels in mutant and WT plants. The total level of eIF4A (SCE) was reduced in the *eif4a1* samples compared with the Col‐0 control, the *eif4a2* levels appeared similar to Col‐0. This was reflected in the IP experiment (eIF4A IP upper panel), less eIF4A protein was affinity purified from the *eif4a1* samples, whereas that from the *eif4a2* samples is similar to Col‐0. In a duplicate experiment (panel below) where more protein was loaded per lane, a smaller band was present only in the *eif4a1* sample.

To gain insight into the role of eIF4A in plant growth, we assessed the publicly available T‐DNA collections for insertional mutants and verified insertions in two GABI‐KAT lines, one for *EIF4A1* (At3 g13920) and one for *EIF4A2* (At1 g54270). The insertion site in the fourth exon of *EIF4A1* was 175 bp downstream of the predicted position, but the predicted position for the insertion in the *EIF4A2* gene was confirmed as correct. A schematic illustration of the insertion sites is shown in Figure 1a.

To evaluate how the T‐DNA insertions affected gene expression, we used RT‐PCR to compare transcript levels and integrity with wild‐type plants (Col‐0 ecotype) and western blotting to compare protein levels. Intact *EIF4A1* mRNA was not detectable (using primers that span the T‐DNA insertion site) from *eif4a1* homozygous plants (Figure 1b, *EIF4A1*). When primers 1 and 3 were used for RT‐PCR, a product was detected (Figure 1b, *EIF4A1* T‐DNA), indicating that a partial transcript is produced and possibly can be translated to produce a truncated protein of approximately 28 kDa. Similar experiments on the *eif4a2* homozygous plants indicate that these are complete knock‐outs at the transcript level, as no *EIF4A2* transcript could be detected (Figure 1b, *EIF4A2* and *EIF4A2* T‐DNA). In *eif4a1* mutants, eIF4A protein levels are reduced and a smaller protein fragment (~30 kDa) is present, these features are not seen in the *eif4a2* mutant (Figure 1c,d).

Homozygous *eif4a1* and *eif4a2* plants were crossed to obtain double mutants; however, from a total of 452 F_2_ plants derived from such crosses, no double homozygous mutants were recovered (Table 1). Certain genotypes were over‐represented in the F_2_ population: for example, the genotypes *EIF4A1*/*EIF4A1–EIF4A2*/*EIF4A2*,* EIF4A1*/*EIF4A1–EIF4A2*/*eif4a2* and *EIF4A1*/*eif4a1–EIF4A2*/*EIF4A2* were recovered more frequently than expected, whereas *EIF4A1*/*eif4a1–EIF4A2*/*eif4a2* individuals were slightly rarer than expected. Genotypes *EIF4A1 eif4a1–eif4a2 eif4a2* and *eif4a1 eif4a1–EIF4A2 eif4a2* were severely under‐represented. Selfed seed from an *EIF4A1 eif4a1–eif4a2 eif4a2 *F_2_ plant was sown and 127 F_3_ plants were genotyped, but again double homozygous individuals were not detected (Table 2). This confirmed that the *eif4a1 eif4a2* double homozygous mutant could not be recovered, and is most likely to be lethal.

**Table 1:** Genotyping data indicating the segregation ratios from 452 F_2_ plants from a cross between *eif4a1* and *eif4a2* parent plants

Expected ratio	Genotype	Number of plants		Observed ratio
	4A1/4A2	Expected	Observed	
1	++/++	28.25	57	2.02
2	++/+−	56.50	117	2.07
1	++/− −	28.25	40	1.42
2	+−/++	56.50	113	2.00
1	− −/++	28.25	23	0.81
4	+−/+−	113.00	100	0.88
2	− −/+−	56.50	8	0.14
2	+−/− −	56.50	6	0.11
1	− −/− −	28.25	0	0

The observed ratio was calculated as the observed number of plants divided by the expected number of plants.

**Table 2:** Genotyping data indicating the segregation ratios of 127 F_3_ plants from two F_2_ plants identified as (+−/− −)

Expected ratio	Genotype	Number of plants		Observed ratio
	4A1/4A2	Expected	Observed	
1	++/− −	31.75	77	2.42
2	+−/− −	63.50	50	0.78
1	− −/− −	31.75	0	0

The observed ratio was calculated as the observed number of plants divided by the expected number of plants.

### *eif4a1* mutants have an ovule abortion phenotype

**Figure 2:**
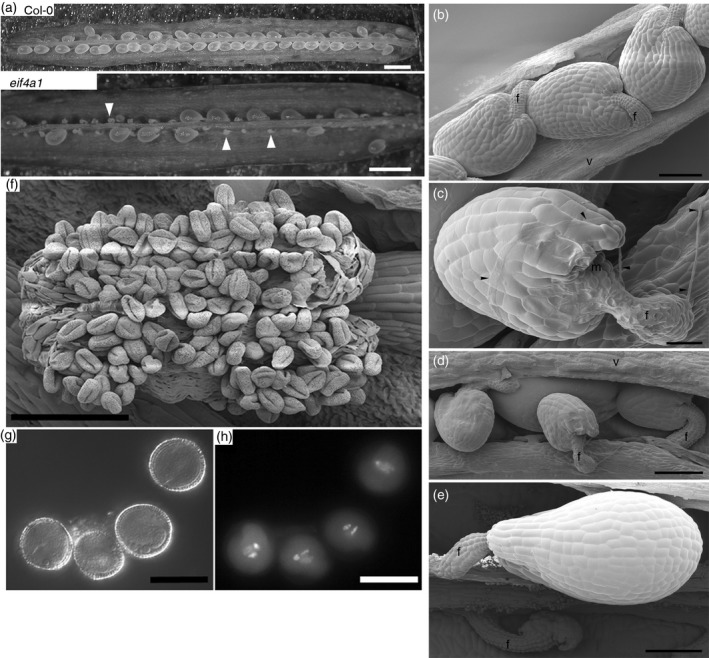
Ovule abortion phenotype of *eif4a1* mutant plants. (a) Dissected Col‐0 and *eif4a1* siliques. (b–e) Scanning electron micrographs of ovules. (b) Col‐0 ovules of uniform size. (c) Pollen tube (arrowed) growing over an *eif4a1* mutant ovule. (d) Three small abnormal ovules overlying a normal ovule in an *eif4a1* mutant silique. (e) An abnormal ovule that has disintegrated and a normally developing ovule in an *eif4a1* mutant silique. (f) A scanning electron micrograph of a dehiscing *eif4a1* mutant anther. (g, h) 4′,6‐Diamidino‐2‐phenylindole (DAPI)‐stained pollen grains seen by Nomarski and epifluorescence microscopy, respectively. Scale bars: (a) 1 mm; (b, d, e, f) 100 μm; (c) 25 μm; (g, h) 15 μm.

To examine the transmission phenotype in more detail, we compared seed set and gamete production in mutant and wild‐type plants. The number of viable ovules was dramatically reduced (by 47–91%) in homozygous *eif4a1* plants compared with Col‐0 (Figure 2a,b,d,e); *eif4a2* homozygous mutants had normal fertility. Scanning electron microscopy indicated that *eif4a1* pollen tubes reached the micropylar region of *eif4a1* ovules (Figure 2c). To determine if the abnormal ovule development was a maternal or a paternal problem, we first examined the anthers and pollen from homozygous *eif4a1* plants. Pollen abundance and dehiscence appeared normal (Figure 2f), and the mutant pollen grains have a well‐defined wall and three nuclei, as seen by Nomarski and 4′,6‐diamidino‐2‐phenylindole (DAPI) staining, respectively (Figure 2g and h). Mutant pollen grains were able to germinate *in vitro* as efficiently as Col‐0 pollen grains (Figure S2a and b), whereas reciprocal crosses between homozygous *eif4a1* and Col‐0 plants (Figure S2c) confirmed the normal transmission of *eif4a1* male gametes. The mean ovule abortion rate in self‐fertilized Col‐0 siliques was 0.6 ± 0.9%, and when emasculated Col‐0 flowers were pollinated with pollen from homozygous *eif4a1* flowers this rose slightly to 1.8 ± 2.5%. If Col‐0 pollen was used to pollinate emasculated *eif4a1* flowers, the abortion rate rose dramatically to 77.6 ± 15.0%, slightly higher than that of selfed *eif4a1* plants (67.2 ± 15.4%). This indicates that a maternal deficiency is responsible for the observed ovule abortion in the *eif4a1* mutant.

### Ovule abortion is associated with nucellar extrusion, followed by ovule degradation

**Figure 3:**
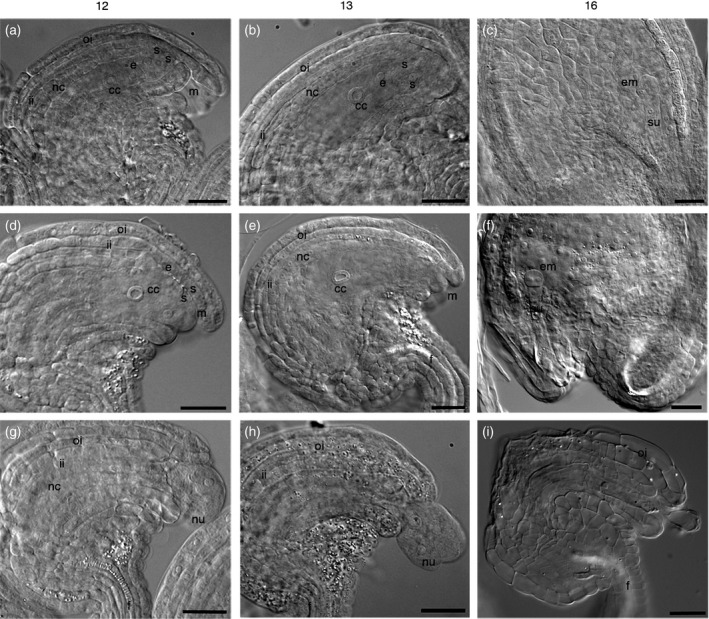
Abnormal ovule development in *eif4a1* mutant plants. (a–i) Nomarski microscope images of ovules from Col‐0 and *eif4a1* mutants at different stages of floral development (stages 12–16; Smyth *et al*., 1990). (a–c) Col‐0 ovules showing normal development. (d–f) *eif4a1* mutant ovules developing normally. (g–i) *eif4a1* mutant ovules developing abnormally. (a, d) In normal development, the integuments surround the nucellus. (g, h) The nucellus in some *eif4a1* ovules is extruded from the embryo sac. (b, e) The ovule enlarges slightly, and the micropyle curves around towards the funiculus. (c, f, i) After fertilization, embryos can be seen in normally developing ovules, whereas some *eif4a1* ovules show tissue degradation. Abbreviations: cc, central cell; e, egg cell; em, embryo; f, funiculus; ii, inner integument; m, micropyle; nc, nucellar cells; nu, nucellus being extruded; oi, outer integument; s, synergid; su, suspensor. Scale bars: 25 μm.

To determine when ovule abortion occurs we sampled Col‐0 and homozygous *eif4a1* mutant flowers at floral stages 12–16 (Smyth *et al*., 1990), and examined intact ovules by Nomarski microscopy (Figure 3a–i). Up to early stage 12, the mutant pistils bear numerous ovules containing a normal nucellus (with two synergids, an egg cell and a central cell) that are being enveloped by the inner and outer integuments: at this stage it is not possible to determine which ovules are going to abort and which will develop normally. At late stage 12, when the integuments have enveloped the nucellus in Col‐0 (Figure 3a), some mutant ovules have a normal micropylar region (‘m’ in Figure 3d), but many are abnormal where the nucellus is extruded from the micropylar end of the ovules (‘nu’ in Figure 3g and h). As the anthers dehisce, the embryo sac of some mutant ovules (containing a nucellus of normal appearance) start to enlarge and curve, as observed in wild‐type ovules at stage 13 (Figure 3b, e). Wild‐type and a minority of mutant ovules subsequently develop normally, with a double fertilization event giving rise to the embryo and endosperm (Figure 3c, f); however, the majority of mutant ovules lose their nucellus, and these embryo sacs appear empty. These aberrant ovules also curve and enlarge slightly, so that the micropyle becomes positioned close to the funiculus and nucellar extrusion can be observed up until stage 14, but further ovule development is suspended. The aberrant ovules collapse during or after stage 15–16 (Figure 3i). If fertilization is prevented by emasculation, collapse does not occur and ovules remain viable for several days. These observations suggest defective coordination of cell and/or organ size during ovule development.

### Reduction in eIF4A‐1 levels perturbs meristem activity

**Figure 4:**
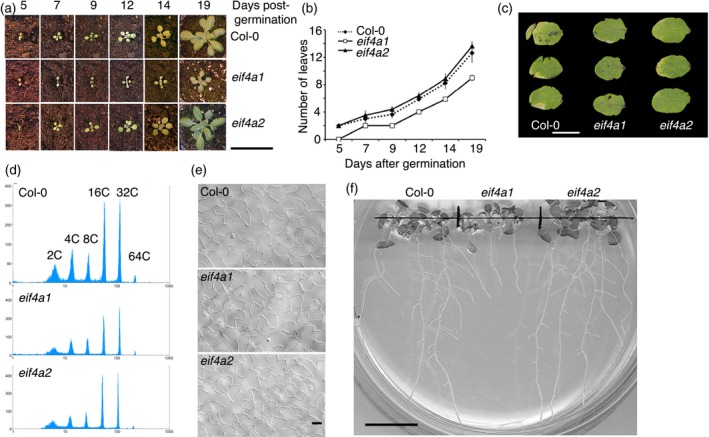
Phenotype of eif4a1 mutant plants. (a) Photographs of Col‐0, *eif4a1* and *eif4a2* plants taken at different stages of vegetative growth. (b) Graph of mean leaf initiation rate after germination. (c) Leaf development in Col‐0, *eif4a1* and *eif4a2*. Images of the adaxial surface of mature fifth rosette leaves sampled 23 days after initiation. (d) Flow cytometric analyses of mature fifth leaves sampled 23 days after initiation from Col‐0, *eif4a1* and *eif4a2* plants. C values are indicated on uppermost graph. (e) Nomarski images of adaxial pavement cells of mature fifth leaves sampled 23 days after initiation from Col‐0, *eif4a1* and *eif4a2* plants. (f) Root morphology in Col‐0, *eif4a1* and *eif4a2* seedlings grown vertically on phytagel for 9 days. The *eif4a1* roots are short and lack lateral roots. Scale bars: (a) 50 mm; (c) 10 mm; (e) 50 μm; (f) 20 mm.

To evaluate the role of eIF4A in vegetative development, we analysed the shoot phenotype of homozygous mutants for each gene. During the early vegetative growth phase, homozygous *eif4a1* plants show an approximately 5‐day developmental lag compared with either Col‐0 and homozygous *eif4a2* plants, between which there is no significant difference (Figure 4a). Homozygous *eif4a1* plants produce leaves more slowly (0.47 leaves per day), compared with Col‐0 and homozygous *eif4a2* plants (0.66 leaves per day), over the first 19 days of growth on soil (Figure 4b). Shoot meristem activity, therefore, is retarded by the loss of eIF4A‐1. Leaf growth, however, is almost normal. We carefully examined final leaf morphology between Col‐0 and the two eIF4A mutants by measuring mature fifth rosette leaves from each line (Figure 4c). Analysis of the digital images (Table S2) showed minor differences in leaf blade area, length, width, perimeter and shape between the three plant lines, but these were not significant, as judged by Student's *t*‐test. Flow cytometric analysis of mature fifth leaves indicated that the spectrum of ploidy levels did not differ greatly between mutant and wild‐type cells (Figure 4d; Table S3). Moreover, trichomes on mutant leaves had normal wild‐type morphology, and the pavement cell density of the adaxial surfaces of *eif4a1* and *eif4a2* fifth leaves was similar (Student's *t*‐test *P* = 0.928; Figure 4e). The dynamics of leaf growth are also similar in all genotypes, as judged by measuring the fifth leaf at different stages of development (Figure S3), indicating that neither *eIF4* gene is essential for leaf growth and that both are likely to be functionally redundant at this stage.

Root growth was also perturbed in homozygous *eif4a1* plants (Figure 4f). Over a 9‐day period, the roots of *eif4a1* seedlings grew less than 20 mm and were devoid of lateral roots, whereas the roots of the Col‐0 and *eif4a2* seedlings were up to 3.5 times longer and bore numerous lateral roots.

### Complementation rescues the mutant phenotypes

**Figure 5:**
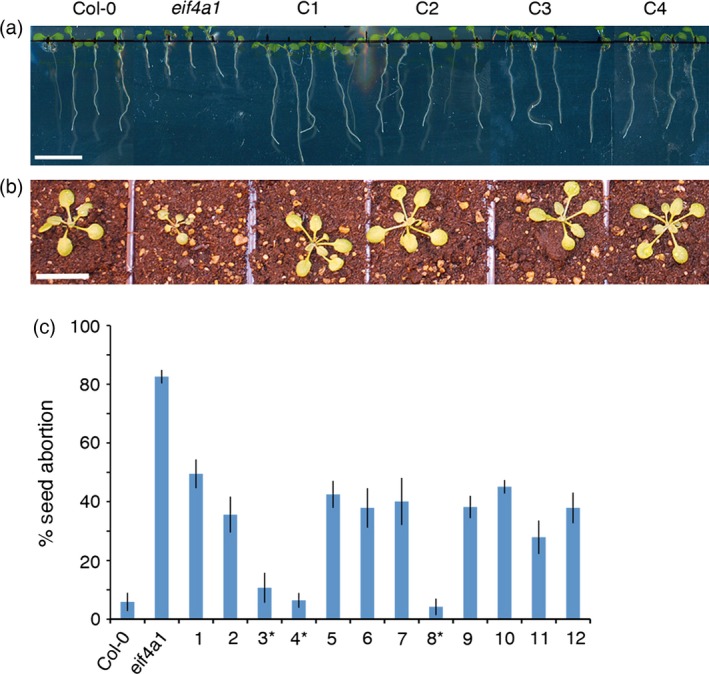
Functional complementation of the *eif4a1* mutant. (a) Complementation of the root phenotype in *eif4a1* with a genomic copy of *EIF4A1*. From the left, Col‐0 seedlings, *eif4a1* mutant seedlings and four independent lines (C1–C4) of *eif4a1* mutants complemented with *EIF4A‐1(p)::EIF4A‐1*. (b) Functional complementation of the aerial phenotype. Plants are labeled as in (a). (c) Complementation of the ovule abortion phenotype. From the left, Col‐0, *eif4a1* mutant and 12 independent complemented lines of the *eif4a1* mutant (1–12); *lines homozygous for the complemented construct. Scale bars: (a) 10 mm; (b) 20 mm.

To confirm that the phenotypes described were caused by the T‐DNA insertion in the *EIF4A1* gene, we introduced a wild‐type genomic copy of the gene driven by its own promoter into the mutant, and assessed the growth and fertility phenotypes of 12 independent transgenic lines. All 12 were essentially wild type in terms of root growth (Figure 5a), and when transferred to soil, all of the complemented lines grew at a similar rate to Col‐0 (Figure 5b), flowered with normal or nearly normal timing, and had reduced ovule abortion (Figure 5c). Abortion in three lines (homozygous for the complemented wild‐type gene) was reduced to wild‐type levels, whereas abortion in nine other lines was reduced to 40–66% (compared with the *eif4a1* mutant abortion rate of 82.6%). The rescued phenotype was stably transmitted in all complemented lines.

### Loss of eIF4A does not affect root histology or meristem size

The ovule phenotype strongly suggested that eIF4A could have a more general role in either cell size control or tissue organisation. An examination of the thick resin sections of Col‐0 and *eif4a1* mutant root tips revealed no gross defects in tissue organisation or meristem size (as judged by the distance from the quiescent centre to the beginning of the zone of cell elongation), compared with the wild type (Figure S4).

**Figure 6:**
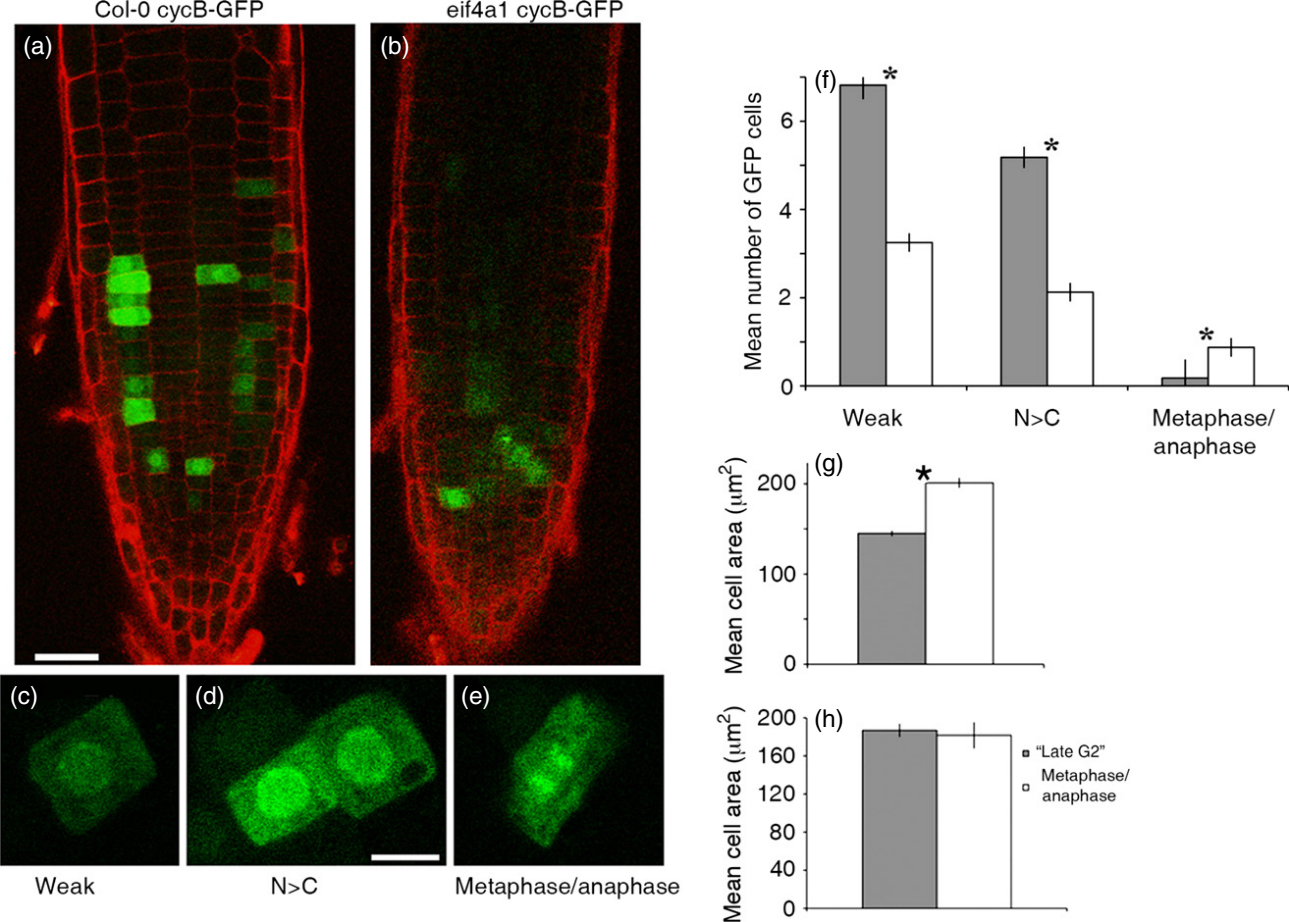
Confocal scanning microscope images of root tips expressing a cyclin B1‐1::GFP reporter. (a) Col‐0 and (b) *eif4a1* roots. (c–e) Cells expressing different patterns of cyclin B1‐1::GFP as they progress through mitosis; N > C indicates stronger nuclear labelling compared with the cytoplasm; metaphase/anaphase includes cells where the chromatin is stained and aligned on the metaphase plate or has separated. (f) Histograms showing the mean numbers of cyclin B::GFP‐positive root tip cells labelled according to this classification in Col‐0 (grey bars) and *eif4a1* (white bars) backgrounds. (g) Mean cell areas of cyclin B::GFP‐expressing cells from Col‐0 (grey bars) and *eif4a1* (white bars) mutant roots. (h) Mean areas of ‘late G2’ cells (grey bars) and ‘metaphase/anaphase’ cells (white bars) in *eif4a1* roots expressing cyclin B::GFP. Asterisks in (f–h) indicate significant differences according to Student's *t*‐tests. Scale bars: (a) 30 μm, also applies to (b); (c–e) 10 μm.

To evaluate the effect of eIF4A on cell cycle dynamics, we introgressed a cyclin B1‐1::GFP reporter and compared its behaviour in wild‐type and *eif4a1* mutant roots (Figure 6a). Although the reporter transcript contains the 5′‐UTR, translation could be subject to translational modulation by eIF4A. The cyclin gene is expressed in G2 and early M‐phase cells under the control of the DREAM complex (Kobayashi *et al*., 2015), however, and is destroyed in mid‐mitosis by ubiquitin‐mediated proteolysis, probably at the metaphase to anaphase transition (Colon‐Carmona *et al*., 1999; Culligan *et al*., 2006). Therefore the *cyclin B1* gene is under multiple levels of regulation and, combined with cytological analysis, is likely to be a faithful reporter of cell cycle events. The effective size of the actively proliferating region of the meristem, as judged by the mean length of the zone containing GFP‐positive cells, is 18% shorter (111.7 ± 37.3 μm; *n* = 19) in mutant roots than in the wild‐type controls (135.8 ± 26.9 μm; *n* = 11).

### *eif4a1* root tip cells show delayed progression into mitosis

The primary root tips of wild‐type plants showed many more cyclin B1‐1::GFP‐positive cells, as compared with the *eif4a1* mutant (Figure 6), indicating that there is a lower proportion of cells in the G2/M phase of the cell cycle in mutant roots. To assess whether the *eif4a1* mutation affected the size at which cells divide, we compared the area of GFP‐positive cells in mutant and wild‐type roots. The average cell area for GFP‐positive mutant cells was 201.18 μm^2^ (*n* = 136), compared with 145.09 μm^2^ for wild‐type cells (*n* = 198): a 1.4‐fold increase (*P* < 0.001) (Figure 6g). The increased size at G2/M is consistent with delayed entry into mitosis.

Surprisingly, we observed that many of the GFP‐positive mitotic root cells in the *eif4a1* mutant appeared to be in metaphase or even post‐metaphase (Figure 6e). In wild‐type cells, the GFP‐cyclin B1‐1 protein is associated with the mitotic chromosomes, but becomes difficult to detect after metaphase. To characterize this phenotype, we allocated GFP‐positive cells into classes based on intracellular localization: ‘weak’, ‘nucleus brighter than cytoplasm’ (‘late G2’) or ‘metaphase/anaphase’ (Figure 6c,d,e, respectively). In plants expressing cyclin B1::GFP in a Col‐0 background (*n* = 11), there were more weakly expressing cells (6.8 ± 0.32, *n* = 75; Figure 6f), compared with the 24 *eif4a1* roots (3.25 ± 0.21; *n* = 78, *P *< 0.002), more cells with a higher expression in the nucleus (5.2 ± 0.24, *n* = 57), compared with the mutant (2.13 ± 0.21, *n* = 51, *P* = 0.0002), but fewer cells in metaphase (0.2 ± 0.42, *n* = 2), compared with the mutant (0.88 ± 0.21, *n* = 21, *P* = 0.014). In the 24 mutant roots examined from four independent lines, there was no difference in the size of the ‘late G2’ and metaphase/anaphase cells, suggesting that the cell growth had ceased at this point (Figure 6h). These data suggest that the *eif4a1* root tip cells have been additionally delayed in cell cycle progression during mitosis.

### *eif4a1* root tips have fewer, but larger cells in S phase

**Figure 7:**
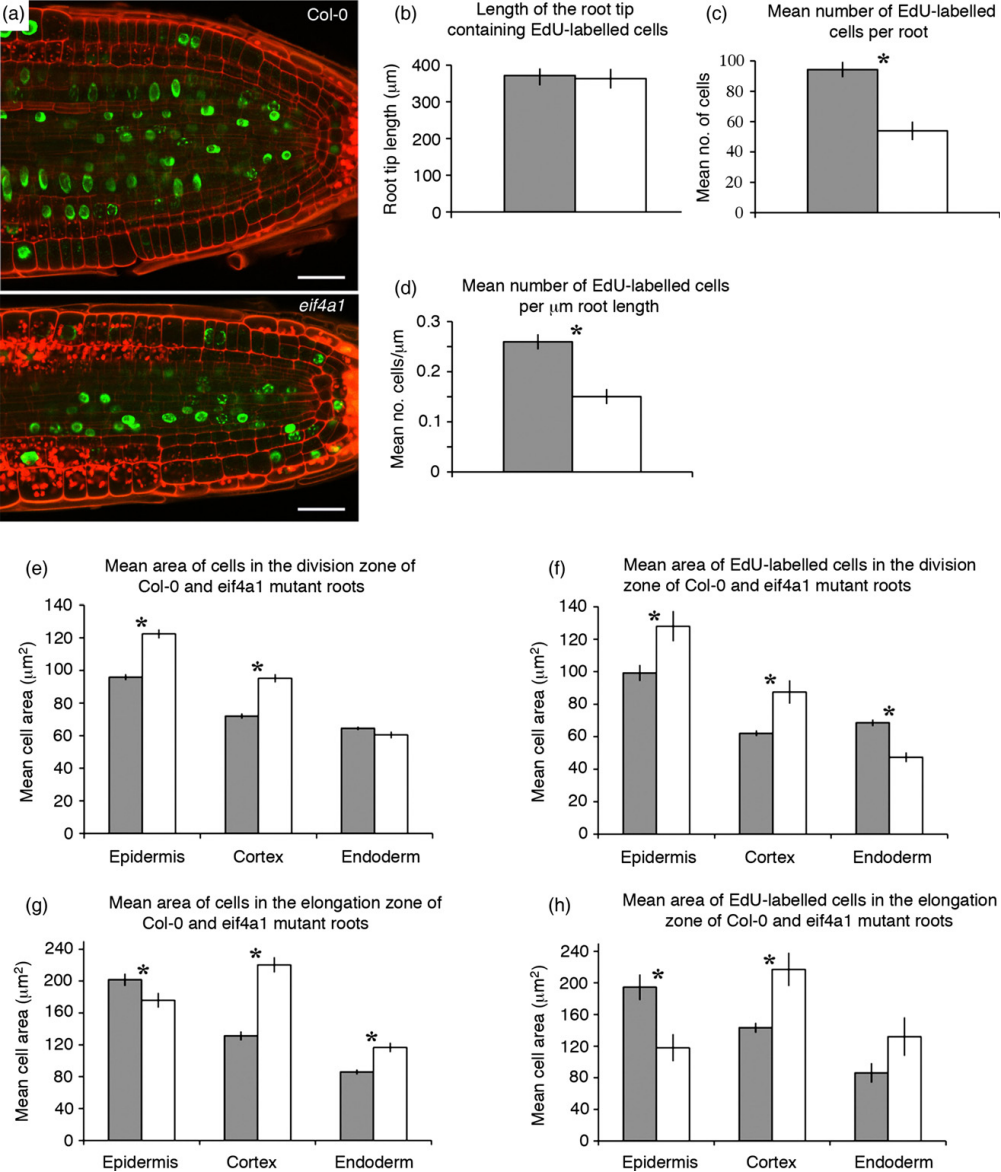
Image analysis data from EdU‐Schiff propidium iodide‐stained and cyclin B::GFP‐expressing roots. (a) Confocal scanning microscope images through the quiescent centre and division zone of EdU‐Schiff propidium iodide‐labelled roots of Col‐0 and *eif4a1* mutants. (b–h) Histograms comparing various parameters of Col‐0 (grey bars) and *eif4a1* (white bars) mutant roots labelled with EdU and Schiff propidium iodide. Asterisks in (c–h) indicate significant differences as shown by Student's t tests.

To directly assess how *EIF4A1* affected cell cycle events in the root, we treated seedlings with ethynyl deoxyuridine (EdU), a thymidine analogue that can easily be detected in plant tissues using Click‐iT® chemistry to couple the EdU with a fluorochrome (Kotogany *et al*., 2010), followed by a modified pseudo‐Schiff propidium iodide staining technique (Figure 7a). In both Col‐0 and *eif4a1* mutant seedlings (*n* = 11 for each) with roots of 5–7 mm in length, the EdU‐labelled cells occupied a zone of similar length (371 ± 74 and 363 ± 84 μm, respectively; Figure 7b). The mean number of EdU‐labelled cells in root tips (in the plane of the quiescent centre) was much lower in the mutants (54.9 per root), however, compared with 94.33 cells in Col‐0 roots (Figure 7c; *P* < 0.001). This equated to 0.26 ± 0.06 labelled cells per μm of Col‐0 root, compared with 0.15 ± 0.05 labelled cells per μm of a mutant root (Figure 7d). This supports the notion that *EIF4A* function is required for cell cycle progression rather than for meristem size. The cell areas of all epidermal, cortical and endodermal cells in the plane of the quiescent centre of Col‐0 and *eif4a1* mutant roots (*n* = 12 for each) were measured. Mutant epidermal and cortex cells were larger on average than the corresponding wild‐type cells, but endodermal cells did not differ (Figure 7e; *P* < 0.001, see also Figure S4). The same trend was seen for epidermal and cortical cells that were labelled with EdU (Figure 7f; *P* < 0.01), but in this case the labelled Col‐0 endodermal cells were larger than the labelled mutant cells (*P* < 0.001), indicating delayed entry into the S‐phase. In the first 100 μm of the elongation zone, the epidermal cells in Col‐0 roots were larger than the mutant epidermal cells, but the cortical and endodermal cells showed the reverse trend (Figure 7g,h). This indicates that the *EIF4A1* mutation can affect cell size homeostasis at either S‐phase or G2/M, depending on the cell type.

### Cell cycle gene expression is reduced in *eif4a* roots

The reduction in cell cycle activity caused by mutation of the *EIF4A1* gene was confirmed using two additional cell cycle reporters, where the promoter and CDS of the *CDKB2*‐*1* and *CDKB1*‐*2* genes have been translationally fused to GUS (Ishida *et al*., 2009) and also by RT‐PCR (Figure S5a–e). CDKB1 represents a group of CDKs that are expressed during a period from S‐phase through to mitosis, whereas CDKB2‐like genes are expressed in a narrower window at the G2/M transition (Fobert *et al*., 1994). CDKB2, therefore, provides another reporter for mitotic activity within the meristem, whereas CDKB1 expression marks both mitotically active cells and those undergoing S‐phase. Consequently, the *CDKB1‐1*::*GUS* reporter marks a longer region of the root than the *CDKB2‐1*::*GUS* reporter. Mutation of the *EIF4A1* gene reduced both zones and diminished the intensity of staining within the zone. The reduction in the zone marked by CDKB2::GUS expression was more severe than that observed for cyclinB1:1 GFP. The size of the zone marked by CDKB1::GUS expression was more variable in mutant roots than in the wild type, but was strongly reduced in intensity. Transcript levels of several cell cycle genes were investigated by RT‐PCR (Figure S5e). Two S‐phase specific transcripts (HH4 and PCNA) did not vary between Col‐0 and the *eif4a1* mutant, and in contrast to the CDKB2‐1::GUS reporter, neither did those of the two CDKB2 genes; however, both CDKB1 genes and the cyclin B1‐1 transcripts were downregulated in the *eif4a1* mutant roots.

## Discussion

Cell cycle progression is tightly coupled with protein translation in animal cells. Notably, translation is switched off during mitosis by CDK1 phosphorylation and activation of small inhibitory proteins that modulate cap‐dependent translation (Pyronnet *et al*., 2001); however, this regulatory pathway seems to be missing in plants (Pierrat *et al*., 2007), and therefore the regulation of translation during the plant cell cycle remains an open question. *In planta*, the fact that cell size varies between cell types in a characteristic manner during development strongly suggests that the process is tightly regulated. Here we report that eIF4A, one of the most abundant RNA helicases in eukaryotic cells, is essential for plant growth and development, as evidenced by our inability to recover plants null for both *EIF4A1* and *EIF4A2*.

Specifically, the Arabidopsis *eIF4A*‐*1* gene is required for cell size control, and its deletion leads to perturbed ovule development. The female fertility defect caused by reduced eIF4A levels is unusual: ovule collapse occurs upon fertilization, which is associated with pre‐fertilization nucellar extrusion (Figure 3). The nucellar extrusion could result from overgrowth of the female gametophyte or from uncoordinated growth of the different ovule tissues. Extrusion of the megaspore through the micropyle seems to leave the ovule susceptible to lysis, which is promoted by fertilization. The haploid egg cell and its two adjoining synergid cells are located adjacent to the micropyle, and the synergids attract the growing pollen tube, leading to lysis of the pollen tube and release of the male gametophytic nuclei (reviewed by Bleckmann *et al*., 2014). We speculate that pollen tube fusion with the extruded female gametophyte could be the trigger for ovule lysis and death. Reduced eIF4A also leads to a strong reduction in lateral root formation (Figure 4). The formation of lateral roots involves re‐entry of pericycle cells into the proliferation cycle, in response to auxin signals, followed by de novo establishment of a meristem and root emergence (Marhavy *et al*., 2013). The requirement for eIF4A suggests that one or more aspects of this multistep process are modulated at the level of translation.

Although it is the prototypical and founding member of this family of RNA helicases, it is rather unusual in that the eIF4A protein contains only a catalytic domain containing nine conserved motifs found in all members of the family (Andreou and Klostermeier, 2013). Helicase activity of the isolated protein is weak, and transcript unwinding activity depends upon the recruitment of the helicase by specific binding partners, a process that may impart specificity to particular transcripts. The T‐DNA insertion into the *eIF4A1* Arabidopsis gene produces a truncated transcript that would translate into a 28‐kDa protein fragment (Figure 1d). The truncated protein is predicted to consist of conserved motifs up to, and including, the ‘DEAD’ ATPase binding motif II, but lacking the ‘SAT’ motif III involved in ATP‐hydrolysis. Although the role of ATP hydrolysis in unwinding is still disputed (Liu *et al*., 2008), it seems likely that this short protein is non‐functional. We speculated that this fragment could compete with other helicases that might be recruited in an attempt to rescue translation in the mutant, and that the fragment may even conceivably be incorporated into eIF4F complexes to render them inactive; however, the *eif4a1* mutant allele is completely recessive to wild type and probably leads to loss of function rather than a dominant negative or interfering effect, as the heterozygotes appear to be similar to wild type in terms of vegetative growth.

The cellular phenotypes observed in homozygous *eif4A1* mutants are cell type‐specific, but a common feature is an effect on cell size homeostasis. Cells within the proliferative zone of root tips of *eif4a1* mutant plants tend to be larger than the equivalent cells in wild‐type plants. Alternatively, and not exclusively, general protein synthesis could also be inhibited, and this could perturb cell growth; however, a general decrease in protein synthesis might be expected to have the opposite effect, and lead to cells entering the S‐ or M‐phase being smaller than normal cells. The main developmental effect is on meristematic function: both roots and shoots grow slowly (Figures 4 and 5) in *eif4A1* mutants, compared with the wild type, but leaves are of similar size, with equivalent cellular parameters. In our root growth experiments over 9 days, *eif4a1* mutant roots grew more slowly, attained a length 3.5‐fold shorter than the wild type and lacked lateral roots. The reduced growth rate is likely caused, at least in part, by slower cell production, perhaps as a result of delayed progression at G2/M and during the M‐phase. Although the decrease in lateral root formation could also result from defective signalling, closer inspection of cells within the primary root indicates that the point of action is either the S‐phase or G2/M, depending on cell type. In both cases, cell size in the proliferation zone of mutant plants is increased compared with the wild type. As size at mitosis defines the maximum size of these rapidly proliferating cells, this strongly suggests that *EIF4A* affects cell cycle progression, perhaps slowing progression and thereby allowing cells to become larger before entering the M‐phase. This could be an evolutionarily conserved role, as partially functional yeast cdc25 alleles can be suppressed by multiple copies of the yeast *Tif1* (*EIF4A*) gene (Daga and Jimenez, 1999). Point mutations in the yeast cdc25 protein phosphatase cause a delay in G2/M progression, and lead to the production of giant cells. The mechanism of suppression is unknown but increased levels of *EIF4A* could enhance the translation of the partially functional cdc25 protein, or promote the translation of factors that can bypass the reduction of cdc25 function. As higher plant genomes seem to lack a recognizable *cdc25* gene (Boudolf *et al*., 2006; Francis, 2007), *EIF4A* targets for regulation are likely to include other factors involved in cell cycle progression and/or cell growth.

The alternative point of action, the S‐phase, also seems to be conserved, as the size at which *Drosophila* endoderm cells enter the S‐phase is affected by the loss of *EIF4A*. Alleles of the *Drosophila eIF4A* gene specifically affect the S‐phase, leading to a failure to re‐enter the cell cycle during larval development that can be suppressed by over‐expressing the E2F transcription factor (Galloni and Edgar, 1999). E2F regulates the transcription of a variety of S‐phase genes, and its over‐expression restores normal cell cycle progression. Based on this result, the authors speculate that transcripts from regulatory genes such as E2F require higher levels of translation factors than their downstream targets, but other mechanisms cannot be ruled out.

Arabidopsis contains two isoforms of eIF4A that share 97% amino acid similarity. These genes seem to be co‐expressed in growing tissues (see Figure S1), but the phenotypes of the single mutants are quite different. Deletion of *eIF4A*‐*2* in a wild‐type *eIF4A*‐*1* homozygous background does not have a discernable phenotype. In animals there are also two copies, and siRNA‐mediated inhibition indicates that each form has unique functions (Galicia‐Vazquez *et al*., 2012). Galicia‐Vazquez *et al*. (2012) reported that the suppression of *EIF4A1* by siRNA (or hippuristanol) in mammalian cells, which leads to reduced protein synthesis and cell viability, a block on cell proliferation and a twofold increase in eIF4A‐2 protein levels, cannot rescue these phenotypes. Svitkin *et al*. (2001) generated an *eIF4A1* dominant negative mutant (in the PTRELA 1a domain) that inhibited both cap‐dependent and cap‐independent translation of mRNAs with complex 5′‐UTRs. The mutant eIF4A1 protein affected the recruitment of the eIF4F complex to the mRNA cap, but whether there were gene‐specific effects could not evaluated. Gene‐specific functions are also formally possible in Arabidopsis, although the two genes are likely to have resulted from a relatively recent duplication event, as the regions surrounding each gene are also duplicated. Moreover, expression of the Arabidopsis eIF4A1 protein in the monocot, *Brachypodium*, completely repairs the mutant phenotype, despite a slightly lower level of similarity; however, neo‐functionalisation could have occurred since the ancestral gene duplication event, and the interesting question as to whether and the extent to which these genes functionally overlap remains to be evaluated.

Because of the recent identification of mammalian eIF4A as an oncogene drug target (Bhat *et al*., 2015), the extent to which these RNA helicases play a role in differential translation is a particularly pertinent question that is receiving much attention. Mammalian *eIF4* genes are possible oncogenes (Montanaro and Pandolfi, 2004), and the cap‐dependent translational machinery is implicated in the promotion and persistence of certain cancers (Wolfe *et al*., 2014; Stoneley and Willis, 2015). The evidence suggests that mammalian eIF4A1 promotes mitosis in proliferative diseases by converting mRNA complexes (duplexes or quadriplexes) to simplexes, thereby promoting the translation of key oncogenes. Supporting this, eIF4A is a key target of the anti‐proliferative drugs hippuristanol (Bordeleau *et al*., 2006b), pateamine (Bordeleau *et al*., 2006a) and silvestrol (Wolfe *et al*., 2014). *EIF4E* is also highly expressed in cancer cells and, together with *EIF4A*, accelerates and maintains T‐cell acute lymphoblastic leukaemia (T‐ALL) in cultured mammalian cells (Wolfe *et al*., 2014). These studies elucidate the fine detail of mechanisms by which eIF4A could promote translation. Notably, several T‐ALL oncogenes, their associated transcription factors and regulators have transcripts that contain repeated guanine quartet motifs (CGG)_4_ in their UTRs that can form K^+^ ion‐stabilized RNA G‐quadruplex structures. These transcripts are dependent on eIF4A for translation that is sensitive to hippuristanol, pateamine and silvestrol (Wolfe *et al*., 2014). Introducing G‐quadruplex sequences into the 5′‐UTRs of mRNAs normally reduces cap‐dependent translation of transcripts by up to 80%; however, RNA G‐quadruplex sequences are necessary and even essential for the cap‐independent translation of some mRNAs (reviewed in Bugaut and Balasubramanian, 2012).

The Arabidopsis genome contains approximately 43 000 G‐quadruplex sequences with a G_2_ repeat in genic regions that may fold the transcript (Mullen *et al*., 2010) and modulate translation (Kwok *et al*., 2015). Using the Mullen genome‐wide survey of G‐quadruplexes, we found that none of the genes [*CDKB1*‐*1* (At3 g54180) *CDKB2*‐*1* (At1 g76540) and *cyclin B1*‐*1* (At4 g37490)] used as reporters in this study contained these motifs; however, it remains possible that other secondary structures could render mRNA translation subject to eIF4A modulation, as appears to be the case for CDKB2;1. CDKB2;1‐GUS levels are strongly reduced in the eIF4A mutant line compared with the wild type (Figure S5), despite similar levels of transcript.

The direct identification of *eIF4A*‐dependent transcripts has been technically difficult (Mustroph *et al*., 2009), but ribosomal footprinting combined with selective drug treatment provides a route towards this in animal culture cells (Wolfe *et al*., 2014). Transcripts protected by ribosomes only in the presence of functional eIF4A include a number of known oncogenes and two proteins with effects on G2/M transition (CDC25B and CDKN2D, a CDK inhibitor protein). This implies, at least in mammals, that eIF4A does have direct effects on the translation of cell cycle regulators known to influence cell size at mitosis. The identification of the equivalent targets, assuming this mechanism is conserved, could provide insight into the equivalent process in plants. Although plants do not contain a recognizable CDC25 homologue, they do have CDK inhibitor genes (*ICK*/*KRP*) that have strong effects on cell size (Schnittger *et al*., 2003; Cheng *et al*., 2013; Wen *et al*., 2013). Other members of CDK complexes are also candidates. Cyclins that act as positive regulators of CDKs can also control cell size at different stages of the plant cell cycle. For example, D‐cyclins were thought to primarily act during G1/S, but conditional expression experiments showed that they could additionally influence G2/M progression in plants (Koroleva *et al*., 2004). In plants, D‐cyclins comprise a large gene family, some of which are expressed in a tissue‐specific manner and specifically affect cell size in those tissues (Nieuwland *et al*., 2009). Overexpression of cyclin D2 can decrease the average size at which root cells divide (Qi and John, 2007). In yeast, G1 cyclins have long structurally complex 5′‐UTRs that have been implicated in the translational control of expression (Polymenis and Schmidt, 1997). Thus, differential sensitivity of D cyclin translation to eIF4A could explain the variable cell cycle stage at which *EIF4A* affects cell size, but different or additional mechanisms may be involved. Although it is not known if the anti‐cancer drugs are also effective on plants, the mutant lines we describe here should be useful in the experimental identification and verification of eIF4A modulated transcripts in plants.

In conclusion, eIF4A‐1 levels affect cell size homeostasis at different stages of the cell cycle in different cell types. Understanding how eIF4A modulates the translation of specific mRNAs or classes of mRNAs should provide insight into the mechanism by which the cell division cycle is coupled to growth in diverse tissues. These mutants, in conjunction with drugs that perturb protein synthesis, could provide the tools with which to explore this largely unexplored area.

## Experimental Procedures

### Expression of *eIF4A*::GUS fusions in Arabidopsis plants

The *EIF4A1* promoter region (1750 bp upstream of the ATG start codon) was cloned using primers attB1‐*EIF4A1* and attB2‐*EIF4A1*. The *EIF4A2* promoter (1300 bp upstream of the ATG start codon) was cloned with attB1‐*EIF4A2* and attB2‐*EIF4A2*. The sequences in lowercase represent the attB1/2 sites, whereas those in uppercase are specific to the gene (Table S1). The PCR products were moved into the pDONR 207 entry vector via the BP reaction, and then into destination vector pBGWFS7 by the LR reaction, in accordance with the manufacturer's instructions (Invitrogen, now ThermoFisher Scientific, http://www.thermofisher.com). *Agrobacterium* cells (strain GV3101) were transformed with the EIF4A1 and EIF4A2 promoter::GUS plasmids, and Arabidopsis Col‐0 plants were transformed using the floral‐dip method (Clough and Bent, 1998). Plant tissues were fixed in cold acetone for 1 h and incubated in GUS‐staining solution (80 mM NaPO_4_, pH 7.0, 0.4 mM K‐ferrocyanide, 8 mM EDTA, 0.05% Triton X‐100, and 0.5 mg mL^−1^ 5‐bromo‐4‐chloro‐3‐indolyl‐β‐d‐glucuronide) at room temperature (20°C). Chlorophyll was removed with 95% ethanol and samples were observed with a Nikon E800 microscope (Nikon, http://www.nikon.com), with images recorded using viewfinder 3.0.1 (Pixera, http://www.pixera.com).

### RNA and protein analysis

Whole rosettes of 24‐day‐old plants were cleaned of soil, frozen in liquid nitrogen, and then ground to a powder with a pestle and mortar cooled with dry ice. Total RNA was prepared using the Ambion mirVana miRNA isolation kit (Applied Biosystems, http://www.appliedbiosystems.com) and quantified with a Nanodrop 2000 spectrophotometer (LabTech, https://www.labtech.com). Total RNA (3 μg) was treated with 0.5 U DNase 1 (LS 006331; Worthington Biochemical Corporation, http://www.worthington-biochem.com) for 30 min at 37°C, followed by 95°C for 5 min and 0.5 μg total RNA reverse transcribed using the Omniscript RT kit (Qiagen, http://www.qiagen.com) and oligo(dT)12‐18 (Invitrogen), according to the manufacturer's instructions. RT‐PCR was performed for 30–40 cycles using the combinations of primers shown in Table S1. The *APT1* (adenine phosphoribosyltransferase) gene was used as a loading control, and as the primers span five introns enabled the detection of contaminating genomic DNA in the cDNA samples. Protein samples were processed for immunoprecipitation and western blotting with an anti‐wheat eIF4A antibody, as described by Bush *et al*. (2009).

### Plant material, growth conditions and PCR genotyping

The *EIF4A1* (At3 g13920) and *EIF4A2* (At1 g54270) T‐DNA insertion lines in *Arabidopsis thaliana* ecotype Columbia were obtained from GABI KAT (352FO3, *EIF4A1*; 022A11, *EIF4A2*; Kleinboelting *et al*., 2012). For aseptic growth, surface‐sterilized seeds were plated on Murashige and Skoog selection medium containing 0.8% (w/v) phytoagar, 3% (w/v) sucrose and sulfadiazine (S‐6387, 5.25 mg per 1L of medium; Sigma‐Aldrich, http://www.sigmaaldrich.com). Plates were kept in the dark at 4°C for 2 days and then transferred to a growth room at 25°C with continuous light. Plants were grown in a climate‐controlled glasshouse at 20°C (day)/16°C (night), with 16 h of total light between October and March. Genomic DNA was extracted from side shoots using phenol‐chloroform, and the presence of the T‐DNA insertions in *EIF4A1* and *EIF4A2* was identified by PCR genotyping (see Table S3 for the primers used) using primer 3 (in the GABI‐KAT T‐DNA LB), in combination with either of the GABI‐KAT gene‐specific primers (primer 1 for *EIF4A1* or primer 5 for *EIF4A2*). Intact *EIF4A1* genes were identified using primers 1 and 2, and for *EIF4A2*, primers 4 and 5 were used. DNA sequencing of the PCR product obtained using primers 1 and 3 was used to check the insertion point for the T‐DNA in *EIF4A1*. The sequencing data provided 100% accurate and continuous coverage through the T‐DNA genomic insertion point (4 593 122) that was 175 bp downstream of the GABI‐KAT predicted site (4 593 297). The position of the T‐DNA in eIF4A2 was confirmed as 2 026 4467. The PCR product obtained using primers 3 and 5 was of the expected size, and when restriction digested with BstB1 it was cleaved into two fragments of the correct size (345 and 524 bp). EIF4A2 has only one BstB1 cut site. The Genome Centre Facility (http://www.tgac.ac.uk)/John Innes Centre (http://www.jic.ac.uk) performed the DNA sequencing of PCR products.

### Ovule abortion experiments

Seed yield was investigated by dissecting siliques attached to glass microscope slides with double‐sided adhesive tape. Dissected siliques at different developmental stages were flash‐frozen in liquid nitrogen and examined by SEM on a Zeiss Supra 55 VP FEG (http://www.zeiss.com). Duplicated reciprocal crosses between six Col‐0 and six *eif4a1* insertion line plants were performed on emasculated flowers. Mature pollen grains were stained with Partec DAPI staining solution (Partec) and observed using Nomarski and epifluorescence microscopy. *In vitro* germination assays were performed by collecting dehiscing pollen grains from open flowers and incubating them in 24 well plates containing 200 μL of germination medium per well (Lalanne *et al*., 2004). Plates were incubated overnight and pollen tube growth was stopped the following morning by the addition of 4% formaldehyde. More than 5000 Col‐0 and eIF4A‐1 pollen grains were scored for pollen tube growth using an E800 microscope equipped with Nomarski optics. Open siliques were vacuum infiltrated with Carnoy's fixative (three parts 95% ethanol to one part glacial acetic acid, v/v) overnight, hydrated in an ethanol series and cleared in a solution of chloral hydrate, glycerol and water (16 : 2 : 1, w/v/v). Ovules were examined and photographed using a Leica DM6000 microscope (Leica Microsystems, http://www.leica-microsystems.com).

### Phenotyping of the eIF4A T‐DNA insertion lines

For vegetative growth studies, sulfadiazine‐selected homozygous lines of each insertion line were grown together with Col‐0 under glasshouse conditions. Trays of plants were digitally photographed with a Nikon D60 camera every 2 days from the fifth day post‐germination. The fifth leaf from triplicate plants was collected 23 days after initiation, scanned on an HP Scanjet 8200 (Hewlett Packard), with a black velvet background, and the digital images were then used for morphometric analyses using imagej 1.41 (National Institutes of Health, http://imagej.nih.gov/ij). Similarly, triplicate fifth leaves from Col‐0 and the two insertion lines were each chopped in 0.5 mL of Partec extraction buffer to release nuclei, the suspensions were filtered through 30‐μM nylon mesh filters, mixed with 1.0 mL of Partec DAPI staining solution and analyzed on a Partec PAS II flow cytometer (Partec). Cell cycle data were analyzed using flomax (Partec). Triplicate fifth leaves were cleared as described above and adaxial pavement cells were examined and photographed using a Leica DM6000 microscope. The digital images were analysed with imagej.

### Complementation

The eIF4A‐1 T‐DNA insertion was complemented by transforming heterozygous eIF4A‐1 plants with a wild‐type genomic DNA driven by it's own promoter. Heterozygous plants were used because the homozygous plants suffered a severely reduced seed yield. A full‐length genomic DNA fragment of 3199 bp was generated using primers attB1‐flg*EIF4A1*fw and attB2‐flg*EIF4A1*rev, and PFU Ultra II Fusion HS DNA polymerase (Agilent Technologies, http://www.agilent.com). The PCR product was purified with a Gel Extraction Kit (Qiagen), cloned into pDonr207 by the GATEWAY BP Clonase reaction, then into pMDC123 (Curtis and Grossniklaus, 2003) by means of a GATEWAY LR reaction. This plasmid was transformed into competent *Agrobacterium tumefaciens* cells (strain GV3101) by the freeze–thaw method (Holsters *et al*., 1978) and plants were transformed by the floral‐dip method (Clough and Bent, 1998). T_1_ seeds were sown on soil and selected by spraying with d,l‐phosphinothricin (77182‐82‐2; Sigma‐Aldrich), 30 surviving plants were PCR genotyped for the presence of the introduced construct using primers to the Gateway attB1 site (5′‐ACAAGTTTGTACAAAAAAGCAGGCT‐3′) and a primer in the 5′ end of the promoter region (5′‐AATCATGTAATTGTTCCACAAG‐3′), and seeds were collected from 10 PCR‐positive plants. Plants homozygous for both the T‐DNA insertion and the complementing construct were selected with sulfadiazine and d,l‐phosphinothricin, and their growth phenotypes compared with Col‐0 and the original homozygous *EIF4A1* T‐DNA insertion plants.

### Root growth experiments

Columbia‐0 and both *eif4a* T‐DNA insertion mutant seeds were germinated aseptically on Murashige and Skoog media containing 0.5% phytagel in square Petri dishes standing vertically, to allow the roots to grow along the surface. Petri dishes were scanned every day for 4 days and root lengths were quantified using imagej. To investigate the root apical meristem, a cyclin B1:1‐GFP reporter line (Culligan *et al*., 2006) was crossed into the *eif4a1* background. Homozygous *eif4a1* plants expressing the cyclin B1‐1::GFP construct were identified by selection on sulfadiazine and d,l‐phosphinothricin. Seedlings of four such lines and the cyclin B1‐1::GFP parent were grown vertically on phytagel plates and examined by confocal laser scanning microscopy using a Leica SP2 DM IRB inverted microscope with an X60 oil immersion objective. Seed germination was staggered so that the parent and mutant seedlings were of similar size when examined. A *z*‐series of images from the division zone were collected and converted to stacks in imagej. The number of cells expressing cyclin B1‐1::GFP was counted and the length of the division zone measured. Homozygous *eif4a1* plants expressing CDKB GUS constructs (Ishida *et al*., 2009) were identified by selection on sulfadiazine and kanamycin, grown as described for the cyclin B1‐1 marker lines and stained for GUS as described for the promoter *EIF4A*::GUS lines. DNA replication in mutant and wild‐type roots was assessed using ethynyl deoxyuridine (Kotogany *et al*., 2010), followed by a modified pseudo‐Schiff propidium iodide staining of the cell (Schiessl *et al*., 2014). Roots were processed conventionally for embedding in LR White resin, as described by Zheng *et al*. (2014).

## Supporting information


**Figure S1.** *EIF4A* gene expression during plant development.

**Figure S2.** *In vitro* germination assays and reciprocal crosses.

**Figure S3.** Leaf growth morphometry.

**Figure S4.** Sections of resin‐embedded wild‐type and *eif4a*1 roots.

**Figure S5.** CDKB::GUS reporter gene expression in *EIF4A1* and *eif4a1* plants.

**Table S1.** Primers used for PCR genotyping and RT‐PCR experiments.

**Table S2.** Leaf morphometric analyses.

**Table S3.** Flow cytometry data from mature fifth leaves of Col–0, *eif4a1* and *eif4a2* plants.
