# Supplementary figures and images for: The RNA helicase, eIF4A‐1, is required for ovule development and cell size homeostasis in Arabidopsis
Source: Plant J. 2015 Dec 7;84(5):989–1004. doi: 10.1111/tpj.13062 (PMC4737287; doi:10.1111/tpj.13062)

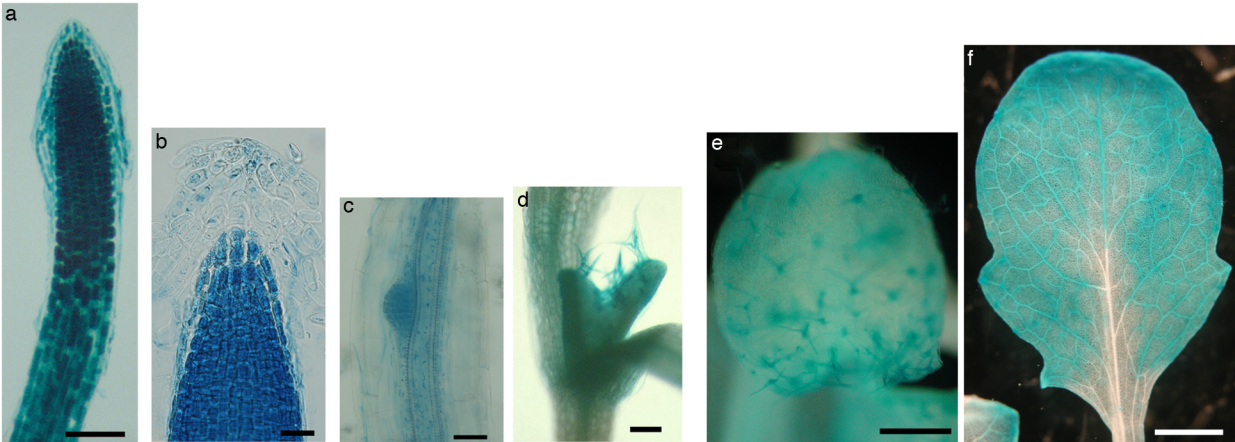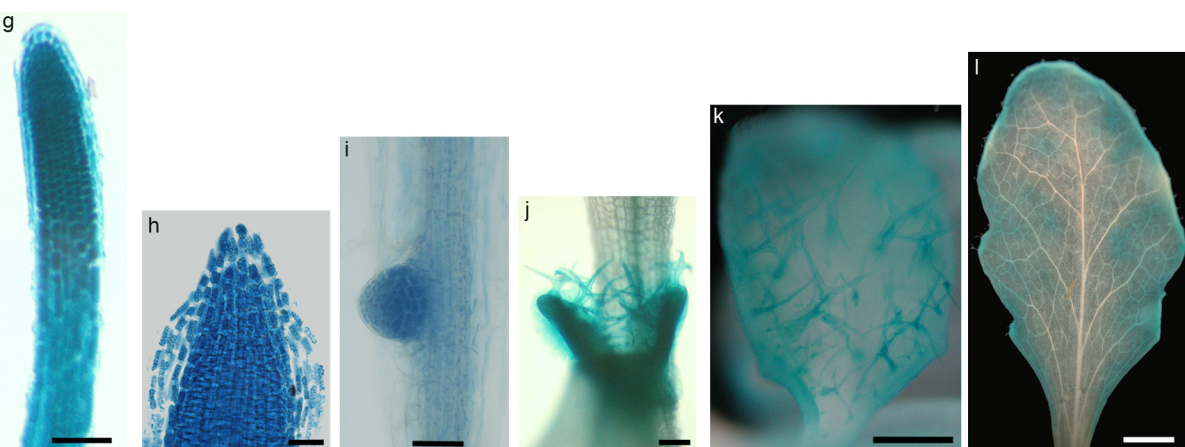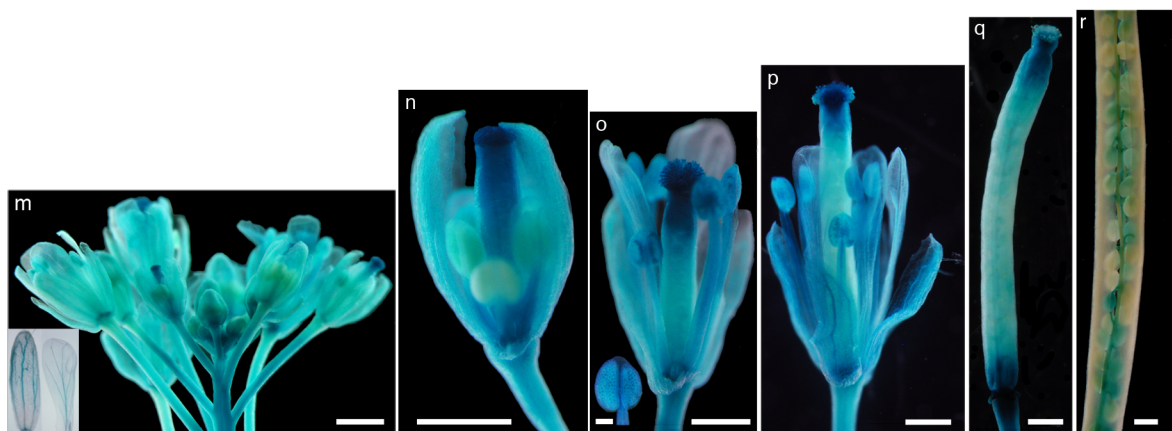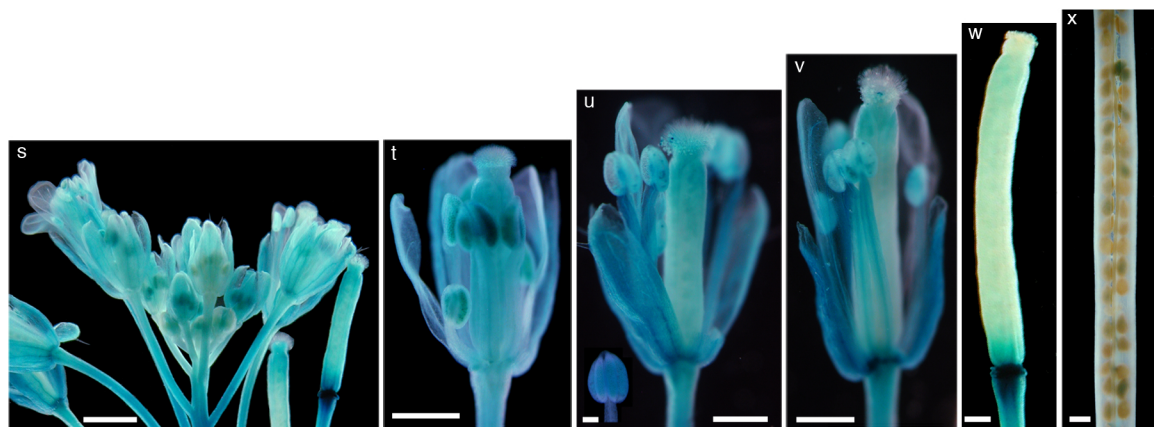

Supplement: Supplementary file 1 — Figure S1. EIF4A gene expression during plant development. [file TPJ-84-989-s001.pdf]

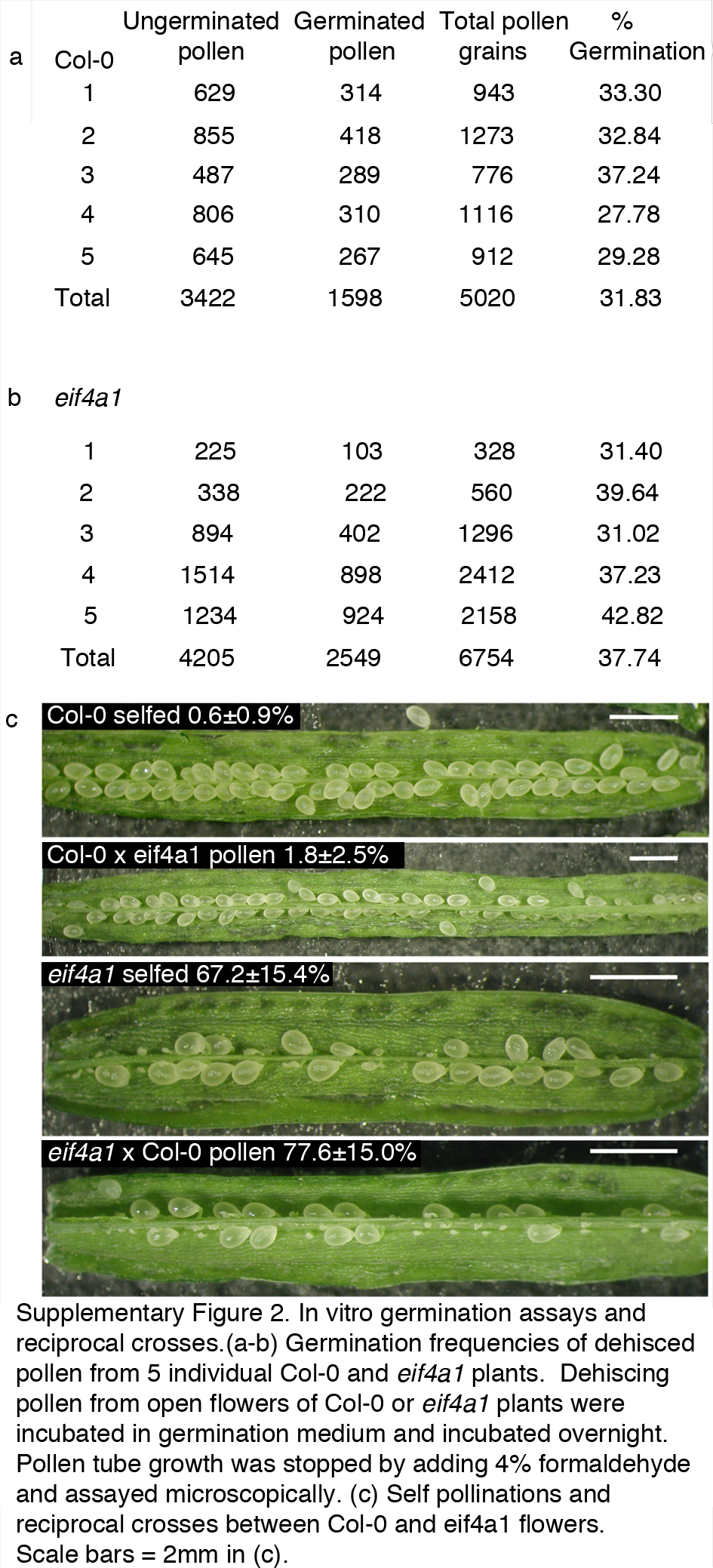

Supplement: Supplementary file 2 — Figure S2. In vitro germination assays and reciprocal crosses. [file TPJ-84-989-s002.tif]

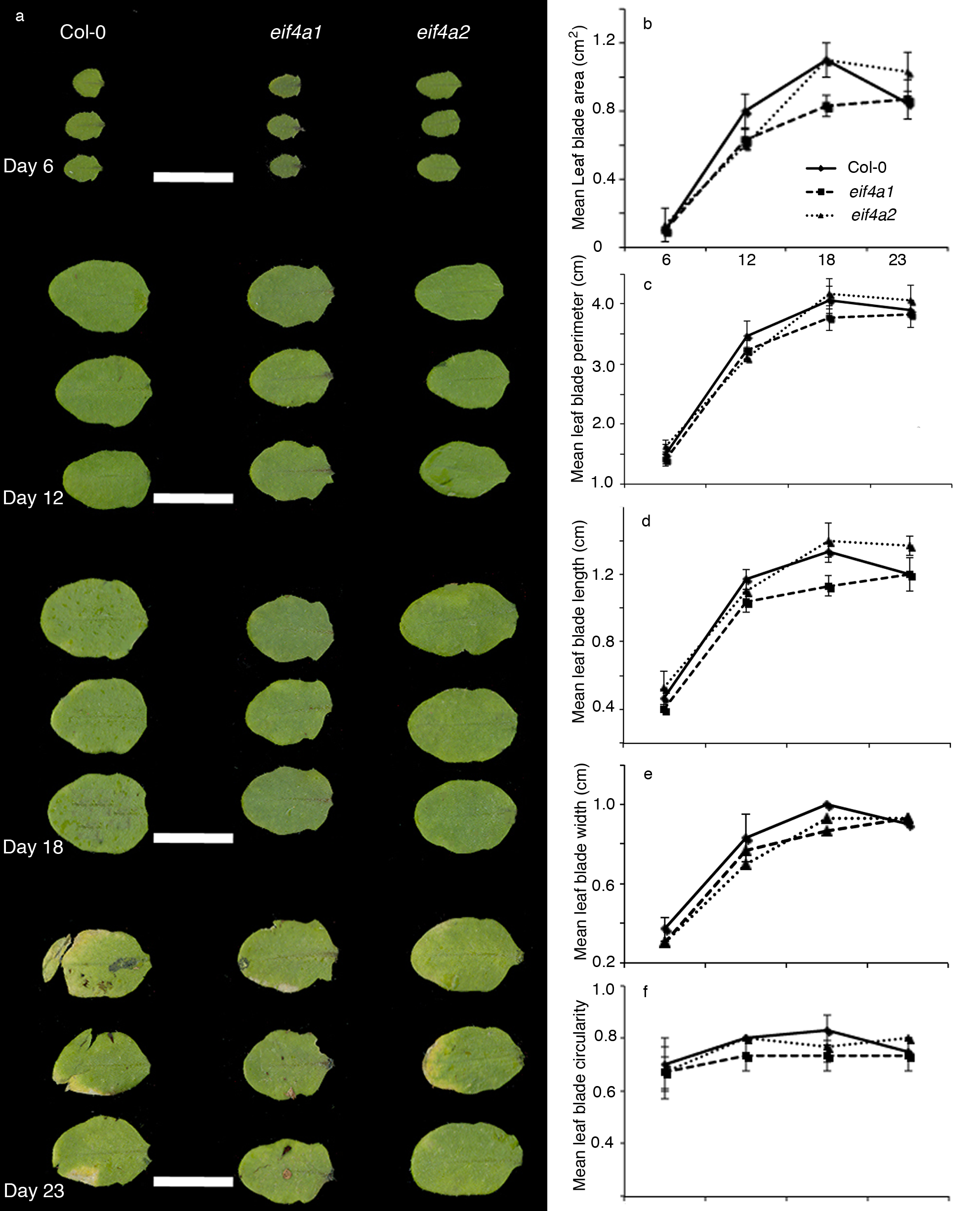

Supplement: Supplementary file 3 — Figure S3. Leaf growth morphometry. [file TPJ-84-989-s003.tif]

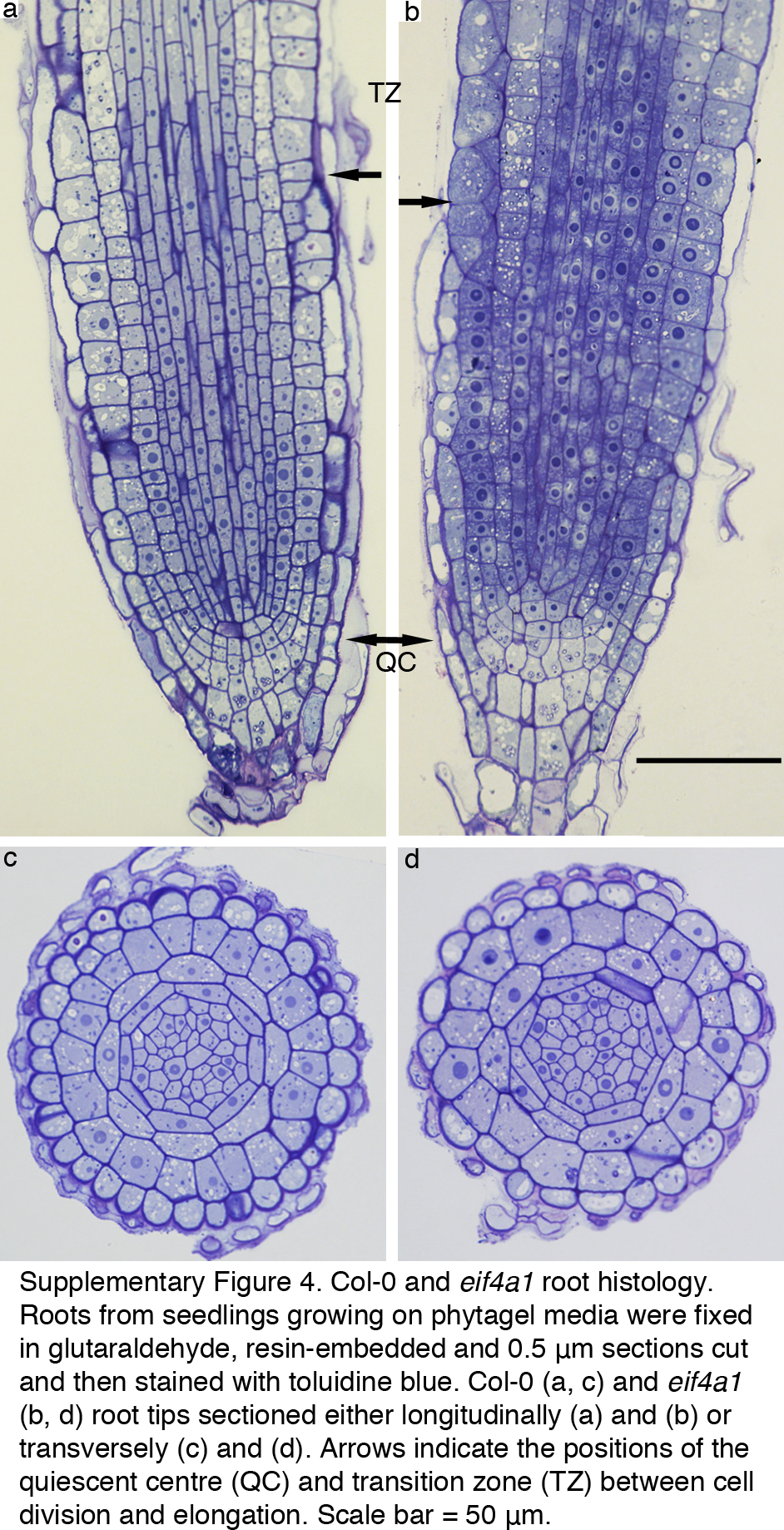

Supplement: Supplementary file 4 — Figure S4. Sections of resin‐embedded wild‐type and eif4a1 roots. [file TPJ-84-989-s004.tif]

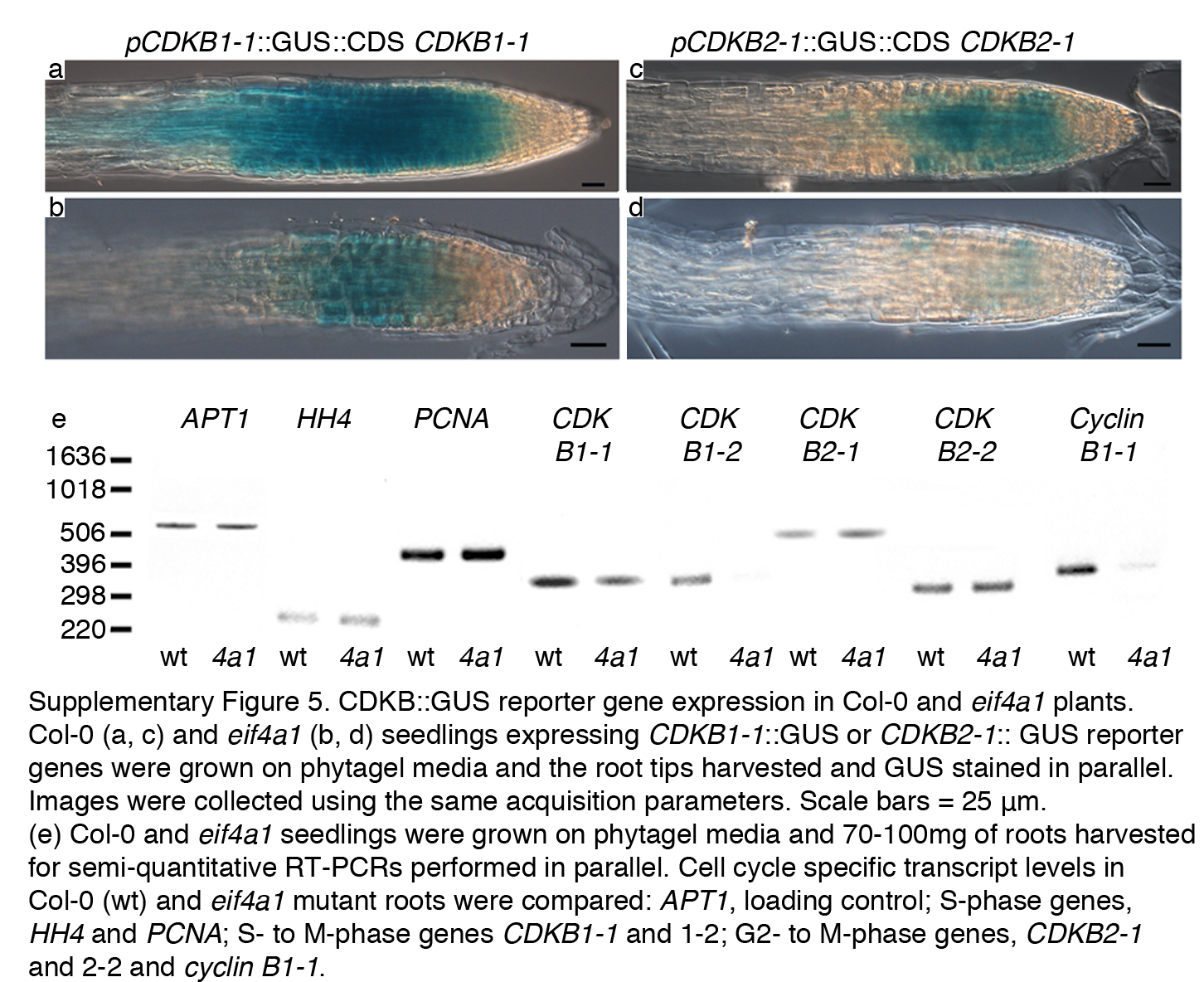

Supplement: Supplementary file 5 — Figure S5. CDKB::GUS reporter gene expression in EIF4A1 and eif4a1 plants. [file TPJ-84-989-s005.tif]
