# Supplementary material for: The RNA helicase, eIF4A‐1, is required for ovule development and cell size homeostasis in Arabidopsis
Source: Plant J. 2015 Dec 7;84(5):989–1004. doi: 10.1111/tpj.13062 (PMC4737287; doi:10.1111/tpj.13062)
Supplement: Supplementary file 6 — Table S1. Primers used for PCR genotyping and RT‐PCR experiments. [file TPJ-84-989-s006.doc]

**Supplementary Table 1. Primers used for PCR genotyping and RT-PCR experiments**

| **Primer name** | **Primer sequence 5’ to 3’** |
| --- | --- |
| 1. GABI-KAT eIF4A-1 forward | GACTTCTCCCTTATCCAGTGTCAG |
| 2. eIF4A-1 reverse | TGGGAGAGAGAGGAGAGAACAACAACTAAGAAA |
| 3. GABI-KAT LB TDNA | CCCATTTGGACGTGAATGTAGACAC |
| 4. eIF4A-2 forward | Gtaagtgaagaaggtttaaag |
| 5. GABI-KAT eIF4A-2 specific reverse | CATAGATCTGCAAATTCACCAGA |
| 6. eIF4A-1 forward | CTTCGATCTCCCAACTCAGC |
| 7. eIF4A-2 reverse2 | AGAATGAAAAAGATGTATCGATGAT |
| 8. APT1 forward | GTGAAATGGCGACTGAAGATGTGC |
| 9. APT1 reverse | GCGACGTCTCTCCTAGTTTCTCCTTT |
| 10. AteIF4A T164A forward | GTGTCCATGTTGTTGTTGGAGCCCCAGGGCGTGTC |
| 11. AteIF4A T164A reverse | GACACGCCCTGGGGCTCCAACAACAACATGGACAC |
| 12. AteIF4A T164E forward | GTGTCCATGTTGTTGTTGGAGAGCCAGGGCGTGTC |
| 13. AteIF4A T164E reverse | GACACGCCCTGGCTCTCCAACAACAACATGGACAC |
| 14. AteIF4A 3’end genotyping | CCAGTTTGACATAAACATATAAAATAAG |
| 15. BASTA forward | GAAGTCCAGCTGCCAGAAAC |
| 16. BASTA reverse | AAGCACGGTCAACTTCCGTA |
| 17. AteIF4A T164A Fw | GTGTCCATGTTGTTGTTGGAGCCCCAGGGCGTGTC |
| 18. AteIF4A T164A Rv | GACACGCCCTGGGGCTCCAACAACAACATGGACAC |
| 19. AteIF4A T164E Fw | GTGTCCATGTTGTTGTTGGAGAGCCAGGGCGTGTC |
| 20. AteIF4A T164E Rv | GACACGCCCTGGCTCTCCAACAACAACATGGACAC |
| 21. AteIF4A 3’end genotyping | CCAGTTTGACATAAACATATAAAATAAG |
| 22. attB1-eIF4A-1 | gtacaaaaaagcaggctacaccaattctaccataaccg |
| 23. attB2-eIF4A-1 | gtacaagaaagctgggtGCTACATAAAAAGAACAAAAACAATCAG |
| 24. attB1-eIF4A-2 | gtacaaaaaagcaggctACCAAGTATCTATCTAGTTTGCTTCAATC |
| 25. attB2-eIF4A-2 | gtacaagaaagctgggtACTACAACAATACACAAAAGAAACAAATAG |
| 26. attB1- flgeIF4A-1fw | ggggacaagtttgtacaaaaaagcaggctACACCAATTCTACCATAACCG-3’ |
| 27. attB2-flgeIF4A-1 rev | ggggaccactttgtacaagaaagctgggttAGGTTTGTGTGGATGTGA |
| 28. eIF4Apeptidefw | ggTTggatccaagcttgtgtccgtgaggatcagcgcatc |
| 29. eIF4Apeptiderev | ggTTgcggccgctcactgcctcttcaacatgtcaaagac |

The sequences in lowercase represent the attB1/2 recombination sites, whilst those in uppercase are specific to the target gene.
