# Supplementary material for: The RNA helicase, eIF4A‐1, is required for ovule development and cell size homeostasis in Arabidopsis
Source: Plant J. 2015 Dec 7;84(5):989–1004. doi: 10.1111/tpj.13062 (PMC4737287; doi:10.1111/tpj.13062)
Supplement: Supplementary file 7 — Table S2. Leaf morphometric analyses. [file TPJ-84-989-s007.doc]

**Supplementary Table 2. Leaf morphometric analyses.**

|  | Leaf blade (mean ± SD) | | | | | |
| --- | --- | --- | --- | --- | --- | --- |
| Plant | Area (cm^2^) | Length (cm) | Width (cm) | Perimeter (cm) | Circularity | Cell density |
| Col 0 | 1.54 ± 0.41 | 1.88 ± 0.25 | 1.11 ± 0.67 | 5.14 ± 0.67 | 0.72 ± 0.03 | 178 ± 14.4 |
| *eif4a-1/eif4a-1* | 1.72 ± 0.29 | 1.84 ± 0.19 | 1.24 ± 0.42 | 5.29 ± 0.42 | 0.77 ± 0.02 | 207.1 ± 41.3 |
| *eif4a-2/eif4a-2* | 1.76 ± 0.2 | 1.82 ± 0.18 | 1.26 ± 0.27 | 5.32 ± 0.27 | 0.78 ± 0.03 | 208.2 ± 26.5 |

Mean measurements of mature 5^th^ leaf blades from Col 0, *eif4a-1/eif4a-1* and *eif4a-2/eif4a-2* plants sampled 23 days after initiation*.* Circularity is an indication of shape where 1 indicates a perfect circle and as the value approaches 0, an increasingly elongated polygon. Cell density refers to the number of pavement cells per mm^2^ of leaf adaxial blade surface.
