# Supplementary material for: The RNA helicase, eIF4A‐1, is required for ovule development and cell size homeostasis in Arabidopsis
Source: Plant J. 2015 Dec 7;84(5):989–1004. doi: 10.1111/tpj.13062 (PMC4737287; doi:10.1111/tpj.13062)
Supplement: Supplementary file 8 — Table S3. Flow cytometry data from mature fifth leaves of Col–0, eif4a1 and eif4a2 plants. [file TPJ-84-989-s008.docx]

**Supplementary Table 3. Flow cytometric data from mature 5^th^ rosette leaves sampled 23 days after initiation.**

| Ploidy level (%) | | | | | | | |
| --- | --- | --- | --- | --- | --- | --- | --- |
| Plant | 2C | 4C | 8C | 16C | 32C | 64C | 128C |
| Col | 23.2 | 16.4 | 18.1 | 25.1 | 15.8 | 1.3 | 0.1 |
| *eif4a-1/eif4a-1* | 20.5 | 18.4 | 14.0 | 24.2 | 20.9 | 1.8 | 0.1 |
| *eif4a-2/eif4a-2* | 19.1 | 16.7 | 13.3 | 31.1 | 18.6 | 1.2 | 0.1 |
