# Supplementary material for: The RNA helicase, eIF4A‐1, is required for ovule development and cell size homeostasis in Arabidopsis
Source: Plant J. 2015 Dec 7;84(5):989–1004. doi: 10.1111/tpj.13062 (PMC4737287; doi:10.1111/tpj.13062)
Supplement: Supplementary file 9 [file TPJ-84-989-s009.docx]

**Supporting Information Legends**

**Supplementary Figure 1.** *EIF4A*::GUS gene expression during plant development.

Col-0 plants were transformed with either p*EIF4A1*:*EIF4A1*::GUS or p*EIF4A2*:*EIF4A2*::GUS constructs and various tissues harvested for GUS staining.

(a-f) and (m-r) p*EIF4A1*:*EIF4A1*::GUS and (g) to (l) and (s) to (x) p*EIF4A2*:*EIF4A2*::GUS constructs in various *Arabidopsis* tissues.

(a, g) Primary roots and their root tips (b, h) express GUS strongly in all cell layers, but this weakens as cells enter the cell elongation zone.

(c, i) Lateral root primordia, arising from the weakly expressing pericyle, upregulate both genes.

(d, j) Shoot apical meristem region showing strong expression in meristems and leaf primordia

(e, k) Trichomes on young leaves.

(f, l) Expanding leaves. Expression first decreases in the basal region (f) and spreads upwards (l).

(m, s) Flowers at different stages of development. Inset in (m) shows a sepal (left and a petal (right).

(n-p) and (t-v) Pistils express GUS more strongly under the *EIF4A1* promoter and GUS expression occurs earlier in pollen under the *EIF4A2* promoter.

(q-r) and (w-x) Late silique and seed development.

Scale bars = 2 mm in (f, l); 1 mm in (m, o, r and s); 500 μm in (n, p, q, t, u, v and x); 300 μm in (e, k and w); 100 μm in (a, d, g, h, inset in o and u); 50 μm in (c, i and j; 25 μm in (b).

**Supplementary Figure 2.** *In vitro* germination assays and reciprocal crosses.

(a-b) Germination frequencies of dehisced pollen from 5 individual Col-0 and *eif4a1* plants. Dehiscing pollen from open flowers of Col-0 or *eif4a1* plants were incubated in germination medium and incubated overnight. Pollen tube growth was stopped by adding 4% formaldehyde and assayed microscopically.

(c) Self pollinations and reciprocal crosses between Col-0 and *eif4a1* flowers.

Scale bars = 2mm in (c).

**Supplementary Figure 3.** Leaf growth morphometry. Fifth leaves from triplicate Col-0, *eif4a1* and *eif4a2* plants were collected 6, 12, 18 and 23 days after initiation, they were scanned on an HP Scanjet 8200 (Hewlett Packard) with a black velvet background (a) and the digital images used for morphometric analyses using ImageJ software. (b-f) Leaf morphometric measurements expressed as mean values plotted against time after leaf initiation, the x-axis and legend applies to all graphs, error bars are the standard error of the mean. The scale bars in (a) = 1cm.

**Supplementary Figure 4.** Col-0 and *eif4a1* root histology. Roots from seedlings growing on phytagel media were fixed in glutaraldehyde, resin-embedded and 0.5 μm sections cut and then stained with toluidine blue. Col-0 (a, c) and *eif4a1* (b, d) root tips sectioned either longitudinally (a) and (b) or transversely (c) and (d). Arrows indicate the positions of the quiescent centre (QC) and transition zone (TZ) between cell division and elongation. Scale bar = 50 μm.

**Supplementary Figure 5.** CDKB::GUS reporter gene expression in *Col-0* and *eif4a1* plants.

Col-0 (a, c) and *eif4a1* (b, d) seedlings expressing CDKB1-1::GUS or CDKB2-1:: GUS reporter genes were grown on phytagel media and the root tips harvested and GUS stained in parallel. Images were collected using the same acquisition parameters. Scale bars = 25 μm.

(e) Col-0 and *eif4a1* seedlings were grown on phytagel media and 70-100mg of roots harvested for semi-quantitative RT-PCRs performed in parallel. Cell cycle specific transcript levels in Col-0 (WT) and *eif4a1* (4A1) mutant roots were compared: APT1, loading control; S-phase genes, HH4 and PCNA; S- to M-phase genes CDKB1-1 and 1-2; G2- to M-phase genes, CDKB2-1 and 2-2 and cyclin B1-1.

**Supplementary Table 1.** Primers used for PCR genotyping and RT-PCR experiments.

**Supplementary Table 2.** Leaf morphometric analyses.

**Supplementary Table 3.** Flow cytometry data from mature 5^th^ leaves of Col-0, *eif4a1* and *eif4a2* plants.
